# Utilisation of an in vivo malaria model to provide functional proof for RhopH1/CLAG essentiality and conserved orthology with *P. falciparum*

**DOI:** 10.1186/s12929-024-01105-7

**Published:** 2025-02-03

**Authors:** Mitchell L. Trickey, Mrittika Chowdury, Georgina Bramwell, Natalie A. Counihan, Tania F. de Koning-Ward

**Affiliations:** 1 School of Medicine, Deakin University, Geelong, Australia; 2 Institute for Mental and Physical Health and Clinical Translation (IMPACT), Deakin University, Geelong, Australia; 3 School of Life and Environmental Sciences, Deakin University, Geelong, Australia

**Keywords:** CLAG, New permeation pathways, *Plasmodium* surface anion channel, Malaria, Gene essentiality, Allelic replacement, Functional conservation

## Abstract

**Background:**

Malaria parasites establish new permeation pathways (NPPs) at the red blood cell membrane to facilitate the transport of essential nutrients from the blood plasma into the infected host cell. The NPPs are critical to parasite survival and, therefore, in the pursuit of novel therapeutics are an attractive drug target. The NPPs of the human parasite, *P. falciparum,* have been linked to the RhopH complex, with the monoallelic paralogues *clag3.1* and *clag3.2* encoding the protein RhopH1/CLAG3 that likely forms the NPP channel-forming component. Yet curiously, the combined knockout of both *clag3* genes does not completely eliminate NPP function. The essentiality of the *clag3* genes is, however, complicated by three additional *clag* paralogs (*clag2, clag8* and *clag9*) in *P. falciparum* that could also be contributing to NPP formation.

**Methods:**

Here, the rodent malaria species, *P. berghei*, was utilised to investigate *clag* essentiality since it contains only two *clag* genes, *clagX* and *clag9.* Allelic replacement of the regions encompassing the functional components of *P. berghei clagX* with either *P. berghei clag9* or *P. falciparum clag3.1* examined the relationship between the two *P. berghei clag* genes as well as functional orthology across the two species. An inducible *P. berghei clagX* knockout was created to examine the essentiality of the *clag3* ortholog to both survival and NPP functionality.

**Results:**

It was revealed *P. berghei* CLAGX and CLAG9*,* which belong to two distinct phylogenetic clades, have separate non-complementary functions, and that *clagX* is the functional orthologue of *P. falciparum clag3.* The inducible *clagX* knockout in conjunction with a guanidinium chloride induced-haemolysis assay to assess NPP function provided the first evidence of CLAG essentiality to *Plasmodium* survival and NPP function in an in vivo model of infection.

**Conclusions:**

This work provides valuable insight regarding the essentiality of the RhopH1 *clag* genes to the NPPs functionality and validates the continued investigation of the RhopH complex as a therapeutic target to treat malaria infections.

**Supplementary Information:**

The online version contains supplementary material available at 10.1186/s12929-024-01105-7.

## Background

During the blood stages of malaria infection, parasites of the *Plasmodium* spp. circulate through the vascular system, invading red blood cells (RBCs) and causing inflammation, leading to several physiological derangements. In 2022, 249 million cases of malaria were reported, of which 608,000 individuals succumbed to the disease, the vast majority of which were children under five [1]. Therapeutic development and deployment have played a critical role in preventing the incidence and severity of this life-threatening disease. Presently, global gains that have been made in combatting malaria are potentially at risk because of the increasing prevalence of delayed parasite clearance following treatment with the current gold standard artemisinin combination therapy [2–4]. It is crucial, therefore, that research continues to investigate novel therapeutic targets that are unique and essential to the *Plasmodium* lifecycle.

The new permeation pathways (NPPs) represent a prospective target for therapeutic development; they are an essential and unique channel(s) exposed on the cytosolic face of the RBC membrane from the trophozoite stage of asexual development [5–8]. The NPPs facilitate nutrient acquisition from the host serum and possibly also aid in the removal of metabolic waste products from the infected RBC (iRBC) [9, 10]. If the NPP is to be considered as a potential drug target, its molecular makeup and structure must be comprehensively understood.

Despite knowing for some time that *Plasmodium*-infected RBCs have an increased permeability attributable to the NPPs [11, 12], the exact structural composition of the channel is still not known [13, 14]. As it stands, the high molecular mass rhoptry protein complex called the RhopH complex, contributes to the formation of NPPs. The RhopH complex is made up of three proteins, RhopH1, RhopH2 and RhopH3, and is uniquely expressed in all *Plasmodium* spp. [15, 16]; it was RhopH1 which was the first to be linked to the NPPs in the most lethal *Plasmodium* spp., *P. falciparum.* [17–20]. Yet, how the RhopH complex, which is secreted from the rhoptry organelles during merozoite invasion, is trafficked to and inserted into the RBC membrane, and what its composition is at this membrane, all remain poorly understood.

Studies have revealed that *P. falciparum* RhopH2 and RhopH3 are encoded by two distinct genes, each essential to parasite survival and NPP activity [21–23]. Conditional gene truncation or depletion of either gene product impacts NPP functionality resulting in parasite death, and interestingly, RhopH3 is also essential to invasion [23]. Conversely, the RhopH1 protein in *P. falciparum* is encoded by five genes (*clag2, clag3.1, clag3.2, clag8* and *clag9*), of which *clag3.1/3.2* have been implicated to encode the channel component of the NPPs [18]. However, knockout of both *clag3* genes shows they are not essential to parasite survival, and *clag3* null parasites only exhibit a decreased growth phenotype when grown in a *Plasmodial* surface anion channel growth inhibition medium (PGIM) that is said to more closely resemble that of human serum [24–26]. Moreover, the *clag3* null parasites show an incomplete reduction in transport of NPP substrates [26, 27]. A conceivable explanation is that the remaining *clag* paralogs complement *clag3* functionality.

All *clag* genes are expressed during the parasite lifecycle, peaking in schizont stage, with varying degrees of epigenetic variation, possibly indicating the expression of distinct NPPs [28–30]. RhopH1 *clag3.1/3.2* expression is epigenetically regulated and undergoes monoallelic expression, where only a single *clag3* gene is transcribed at a time [31, 32]. The switching of expression from one *clag3* gene to the other is potentially in response to host immunity given they are exposed at the RBC surface, or to facilitate the uptake of different nutrients [24, 33]. Like CLAG3.1 and CLAG3.2*,* both CLAG2 and CLAG8 exhibit substantial pairwise diversity across *P. falciparum* spp. in the domain exposed at the host cell surface, which is referred to as the hypervariable region (HVR) [29, 30]. Interestingly, CLAG9 does not contain the same surface exposed variable region [27, 30]. Both CLAG3 and CLAG2 expression can also be suppressed in response to drug pressure, altering the permeability of the host cell to blasticidin, adding an additional level of complexity to how these genes are regulated and expressed [30, 34]. Knockout of either *clag2* or *clag8* has also been shown to significantly increase the transcription of the other *clag* genes, as well as *rhoph2* and *rhoph3*, however, knockout of *clag9* was not shown to have the same effect [27]. Intriguingly, previously published work showed the knockout of *clag3* had a significant impact on *clag9* transcription, but did not affect transcription of any of the other *clag* or *rhoph* genes [26]. Whether different CLAG proteins are present within each RhopH complex at the RBC membrane, or if each membrane complex exclusively contains a single CLAG paralog, resulting in the formation of multiple channel types, remains to be shown.

Previous phylogeny of the CLAG protein family from *P. falciparum* strains of different geographical origins reveal *clag9* sequences are the most divergent of the five *clag* genes [30, 35]. Based on phylogeny of the first and last exon of CLAG proteins from *P. falciparum,* and that of the two CLAG paralogs in *P. vivax* and the rodent species, *P. yoelii,* CLAG members have been grouped into two subgroups, AP and A [29]; each species expresses a single Type AP *clag* gene, represented by *clag9* in *P. falciparum* and a variable number of Type A *clag* genes, encompassing *clag2, clag3.1, clag3.2,* and *clag8* [29, 30, 35–37]. This begs the question as to whether CLAG proteins belonging to different subgroups have a different function. *P. falciparum clag9* gene was in fact the first to be discovered and as it was implicated in cytoadherence, it was hence named cytoadherence linked asexual gene (*clag*) [38, 39]. Knockout of *clag9* was shown to abolish melanoma cell binding and CLAG9 was proposed to be involved in the cytoadherence to endothelial receptor CD36 [40, 41]. More recent studies showed CLAG9 involvement in trafficking of *Pf*EMP1, a surface exposed protein essential to cytoadherence [42], however, another study showed no loss to either *Pf*EMP1 binding or parasite survival following allelic replacement of *clag9* in an in vitro model [43]. CLAG9 has also been implicated in merozoite binding to the RBC surface during invasion, suggesting CLAG9 may have multiple roles throughout the parasite lifecycle [44]. The contribution of CLAG9 to the *P. falciparum* lifecycle, however, is not comprehensively understood but it appears not to be essential for in vitro growth.

Defining RhopH1 *clag* gene essentiality in *P. falciparum* is complicated by the expansion of *clag* genes*.* To definitively test essentiality, several genes would need to be knocked out in a single parasite line which would be practically very time consuming as it would require multiple reiterations of transfection. On the other hand, rodent malaria *P. berghei* contains two RhopH1 genes, simplifying genetic engineering to answer the question of *clag* gene essentiality and complementation. The two *clag* genes in *P. berghei* are a predicted ortholog of *P. falciparum clag9* (*Pb*ANKA_0836300) and a *clag* gene that we herein refer to as *clagX* (*Pb*ANKA_1400600). Importantly, similar to *P. falciparum* RhopH1 proteins, *P. berghei* CLAGX also localises to the merozoite rhoptry bulb with RhopH2 and RhopH3 [45]. Furthermore, parasitised rodent RBCs have been shown to have an increased permeability to a variety of organic compounds [46, 47] that can be blocked by the NPP inhibitor furosemide [48–50]. Despite evolutionary separation, the NPPs have been shown to be functionally conserved across the divergent *Plasmodium* spp., substantiating the examination of rodent NPPs and their relevance to human malaria [15].

Here we conducted a more extensive phylogenetic analysis and examined via allelic replacement whether the *P. berghei clag* genes belonging to distinct phylogenetic clades can complement the function of one another. Secondly, to experimentally determine if *P. berghei clagX* is the functional ortholog of *P. falciparum clag3,* the *P. berghei clagX* sequence encoding predicted transmembrane domains (TMDs), HVRs and critical RhopH2 and RhopH3 binding sites was replaced with that of *Pfclag3.1*. Finally, we examined *P. berghei clagX* gene essentiality and contribution to NPP functionality utilising the inducible diCre-*loxP* system, which provided the first functional proof that the functional orthologue of the *P. falciparum clag3.1* gene is indeed essential to parasite survival and NPP function in vivo*.*

## Methods

### Ethics statement and mice

Female and male ARC(s) Swiss mice (6–10 weeks) or Wistar rats (6 weeks) were sourced from the Animal Resource Centre (Perth, Australia). Rodents were maintained on a standard rodent diet (chow) and housed under controlled conditions at 21 °C with a 12:12 h light: dark cycle. All experiments were approved by the Deakin University Animal Welfare Committee (Project G03-2020 & G03-2023).

### *P. berghei* parasites lines and propagation

Stabilites of RBCs infected with the *Plasmodium berghei* ANKA (*PbA*) strain clone 15cy1 and *PbA* c115cyl HP (854) stored in liquid nitrogen were thawed and injected interperitoneally (IP) into a donor mouse. Experimental mice were infected with 1 $\times$ 10^6^ iRBCs that had been harvested from donor mice. The parasite load in animals was monitored by visualising methanol fixed blood smears stained with Giemsa by microscopy using 100 $\times$ magnification under oil. Cardiac bleeds were performed on infected mice when the parasitemia reached > 5% and when required, parasite lines were preserved by adding 30% (*v/v*) glycerol in mouse tonicity (MT) phosphate buffered saline (PBS) to whole blood at a 1:1 volume, aliquoting into cryovials and freezing in liquid nitrogen.

### In vitro culturing of *P. berghei* parasites and schizont harvest

Infected blood containing ring or trophozoite stage parasites was washed with incomplete RPMI-HEPES culture medium (10.41 g RPMI-1640, 2 g NaHCO_3_, 5.96 g HEPES, 5 ml Neomycin, in 1 L H_2_O). Samples were cultured overnight (~ 16 h) at 3% haematocrit at 36.5 °C in complete culture medium (RPMI-HEPES containing 25% (*v/v*) fetal bovine serum) under 1% O_2_ & 5% CO_2_ in N_2,_ in 30 ml culture dishes until parasites reached schizont stage.

Schizonts were purified as previously described [51]. Briefly**,** cultured schizonts were examined for their morphology by examining Giemsa-stained smears by microscopy to ensure schizont viability and segmentation of merozoites. Cultures were underlayed with 336 mM Iohexol (nycodenz) in buffered medium (5 mM Tris/HCl, 150 mM KCl, 5 mM CaNa_2_EDTA) at a 1:1 ratio with ice cold PBS and spun at 290 $\times$*g* for 20 min at 22 °C using a swing out rotor with slow acceleration and deceleration. Schizonts were visible as a brown ring at the blood/nycodenz interface and were collected and washed with 15 ml of culture media (290 $\times$*g,* 5 min). Pelleted schizonts were resuspended in fresh culture media.

### Extraction of *P. berghei* genomic DNA

Parasite pellets were extracted from mouse RBCs by first collecting whole blood from mice by cardiac puncture, immediately adding ~ 300 U (50 μl) heparin (Hospira). Leucocytes were depleted by filtration through CF-II cellulose powder (Whatman). Blood pellets (500–1000 μl) were added to ~ 2 ml 0.15% (*w/v*) saponin diluted in PBS and incubated on ice for 10 min. Parasite pellets were collected by centrifugation (10,000 $\times$*g*, 5 min, 4 °C) and washed with ice cold PBS three times. 500–700 μl of TNE (50 mM Tris pH 8.0, NaCl_2_, 5 mM EDTA pH 8.0, 1% (*w/v*) SDS, in H_2_O) was used to resuspend the parasite pellet, after which 10 μl of RNAse (Sigma-Aldrich) was added and the sample incubated (37 °C, 30 min). Proteinase K (Invitrogen) was then added to a final concentration of 100 μg/ml and incubated further (37 °C, 45 min). Genomic DNA (gDNA) was extracted by adding an equal volume of 25:25:1 phenol:chloroform:isoamyl alcohol (Sigma-Aldrich), centrifuging (17,000 $\times$*g*, 5 min, room temp (RT)) and then taking the upper phase containing gDNA. Samples were ethanol precipitated by adding 2.5 $\times$ volume of 100% (*v/v*) ethanol and 0.1 $\times$ volume of sodium acetate (3 M, pH 5.2), and then incubated at –20 °C overnight. Samples were spun at > 10,000 $\times$g (30 min, 4 °C) to pellet gDNA. Pellets were washed in 80% (*v/v*) ethanol (> 10,000 $\times$*g*, 5 min, 4 °C), air dried, and resuspended in the required volume of TE buffer (10 mM Tris, 1 mM EDTA, pH 8) for subsequent experiments.

### Generation of transgenic parasite lines

SnapGene Software (version 7.2.1) was used to design oligonucleotides to amplify *P. berghei* or *P. falciparum* genome sequences obtained from PlasmoDB [52] (Release 68, 7 May 2024). Oligonucleotides were synthesised by Sigma-Aldrich. In silico plasmid construct design was conducted using SnapGene software.

Polymerase Chain Reaction (PCR) was used to amplify DNA fragments using the appropriate oligonucleotide pairs listed in Supplementary Table 1. Either purified *Plasmodium* gDNA or pDNA was used as a template for amplification.

The *PbA* 15cy1 line was used to create the *PbclagX* allelic replacement lines (*Pb*_*X/3.1*_*, Pb*_*X/9*_ and *Pb*_*X/3.1extend*_), and their relevant tagging control lines. The *PbA* c115cyl HP (854) marker free diCre line (*Pb*_*diCre*_), kindly gifted by Andrew P. Waters [53], was used to generate the flanked *loxP* (*flox*) *PbclagX* line (*PbclagX/flox*). The *PbA* c115cyl HP line contains a constitutively expressed split Cre recombinase (diCre) expression cassette in the *p230p* locus.

**The pPbX/Pf3.1** construct, contained a 1.3 kb *PbclagX* 5´ homology box (HB) (O1661/O1662) designed upstream and in-frame with the 1 kb *Pfclag3.1* region of replacement. The replacement region was synthesised (IDT) with 3 $\times$ human influenza hemagglutinin (HA) epitope tag appended in frame at the C-terminus. The 5´HB and synthesised fragments were assembled in the pBlueScript-II-KS( +) (pBlueScript) construct, utilizing 5´*Apa*I, 3´*Sac*II and a native *Cla*I enzyme site in the *PbclagX* locus. The complete in-frame fragment with HA tag was then removed from the pBlueScript construct using *Apa*I and *Nae*I enzyme sites, and consequently ligated into the p35 construct [54]. A *Sal*I site was inserted prior to the first HA sequence to facilitate the assembly of subsequent constructs. In the p35 construct, the *PbclagX/Pfclag3.1* region was terminated with a *cam-*3´ untranslated region (UTR) (O1651/O1652) that was ligated into the plasmid using *Nae*I and *Sac*II sites. The p35 plasmid contains a human *dihydrofolate reductase* (DHFR) drug resistance cassette with an *ef1α-*5´ promotor and DHFR 3´ transcription terminator sequence (DT-3´), both derived from *P. berghei* [54]. A 3´ HB was generated from the *PbclagX* 3´ UTR by PCR (O1649/O1650) and ligated into the construct using *Kpn*I and *EcoR*V enzyme sites. Following the initial p*PbX/Pf3.1* construct assembly, only the 5´ targeting and replacement region needed to be switched out for the following replacement constructs using *Apa*I and *Sal*I.

**The pPbX/3.1cntrl** construct replaced the 1 kb *Pfclag3.1* sequence in the pBlueScript construct with recodonised *PbclagX* sequence, using *Cla*I and *Sal*I sites then removed using *Apa*I and *Sal*I and ligated into p35 construct.

**The pPbX/9** construct was assembled with a 1.6 kb *PbclagX* 5´HB (O1888/O1889) sewn together with in-frame 1.4 kb *Pbclag9* replacement sequence (O1890/O1868). The sewn fragment was ligated into the p35 construct using *Apa*I and *Sal*I sites.

**The pPbX/9cntrl** construct contained the 1.6 kb *PbclagX* 5´HB (O1935/O1936) sewn together with synthesised recodonised *PbclagX* sequence (GenScript) and ligated into the p35 construct using *Apa*I and *Sal*I sites.

**The pPbX/3.1extend** construct used 1.2 kb *PbclagX* 5´ HB (O1981/O1982) assembled in pBlueScript together with 2.4 kb *Pfclag3.1* (O1983/O1984) aligned sequence. The amplified 5´ HB was ligated into pBlueScript first using *Apa*I and *Xho*I sites, following this the *Pfclag3.1* sequence was ligated in using native *Mfe*I and *Sal*I enzyme sites. The complete fragment was removed by *Apa*I and *Sal*I and ligated into the p35 construct.

**The pPbclagX/flox** construct was designed to *flox* the last 1200 bp (400 amino acids (aa)) of the *P. berghei clagX* gene. To assemble this construct, a fragment was synthesised by GenScript, which comprised of the following: a ~ 750 bp 5´ HB that lies immediately upstream of the *flox* region, followed by an intron containing the 5´ *loxP* sequence and then a recodonised version of the 1200 bp region, followed immediately by a 3 $\times$ cMyc epitope tag, stop codon, and finally the 3´ *loxP* sequence. The entire fragment was delivered in the pBlueScript plasmid. The enzymes *Apa*I and *Sal*I were used to remove the complete fragment from this plasmid and the fragment was subsequently ligated into the p35 construct using *Apa*I and *Sal*I enzyme sites.

**The CRISPR-Cas9 (pABR099)** constructs [55] were used in transfection to improve efficiency of recombination and contained a single-guide RNA (gRNA) sequence for the relevant *clag* gene. The gRNAs were designed upstream of a Protospacer Adjacent Motif (PAM) sequence to enable recognition for CRISPR/Cas9 genome editing by DNA cleavage using the online platform ‘Benchling’ and publication by Doench, Fusi [56]. For each transfection, two gRNA sequences were generated by PCR (*PbX/Pf3.1;* gRNA1 O1723/O1724, gRNA2 O1725/1726, *PbX/9* and *PbX/flox;* gRNA1 O1915/O1916, gRNA2 O1917/1918, *PbX/Pf3.1extnd;* gRNA1 O1991/O1992, gRNA2 O1993/1994) and ligated into pABR099 using *Esp3*I enzyme site. The region in the targeting constructs containing gRNA targets were either replaced or recodonised to inhibit the continuous cleavage of the locus once the construct had integrated.

### Transfection of *P. berghei*

pDNA was transfected into schizont-stage parasites according to an established protocol [51]. Briefly, 3–6 μg of pDNA constructs for transfection were linearised with the appropriate restriction enzyme overnight, precipitated, and resuspended in 10 μl of TE. 100 μl of Amaxa nucleofector (Lonza) containing pDNA was added to Nycodenz purified schizonts and electroporated using an Nucleofector II (Amaxa biosystems) device and program U33 according to manufacturer instructions. Schizonts were transferred to an Eppendorf tube and 50 μl RPMI media added, samples were then drawn up into a 1 ml insulin (29-gauge) syringe and injected intravenously (IV) into 5–6-week-old female Swiss mice. Transfectants were selected by administering 70 μg/ml pyrimethamine (pyr) (pH 4.5) to the drinking water of mice ~ 24 h after transfection. Tail blood smears were made just prior to drug administration and then again from day seven post-transfection and stained with Giemsa to determine if parasites survived the selection. Once the parasitemia of infected mice reached minimum 5%, the mice were humanely killed by slow fill CO_2_ and blood harvested. The *Pb*_*clagX/loxP*_ parasite line was cloned out by limiting dilution, where Giemsa stains were used to calculate parasitemia and haematocrit to calculate iRBCs/ml. Subsequently, dilutions were made using PBS so that each mouse was injected IV with one parasite using a 29-gauge insulin syringe.

### Genome editing of diCre-*loxP* line

The induction of *loxP* recombination in clonal *PbclagX/flox* parasites using rapamycin was performed both in vitro and in vivo. A stock solution of rapamycin (Invitrogen) was made in Dimethyl sulfoxide (DMSO) at 4 mg/ml. For in vitro induction, *PbclagX/flox* parasites were harvested from infected mice and cultured in complete culture medium containing either 200 nM rapamycin or vehicle control (DMSO) to schizont stage. Parasite pellets were harvested as described above for gDNA and protein analysis. For in vivo induction, five donor mice were infected IP with *PbclagX/flox* and monitored until parasitemia reached ~ 5%, after which iRBCs were collected and cultured to schizonts and nycodenz purified. Ten mice were then injected IV with ~ 1 $\times$ 10^8^ purified schizonts, and ~ 24 h later 5 mice were IP injected with 4 mg/kg of rapamycin (+ Rap), to induce excision, whilst the remaining 5 mice were treated with vehicle control (–Rap). Parasites were monitored via tail smear at various time points and harvested via cardiac puncture for gDNA and protein analysis.

### Western blotting analysis

Cultured *P. berghei* pellets were obtained using the same lysis protocol as the gDNA extraction. Additionally, 2% (*w/v*) Roche cOmplete protease inhibitor cocktail (Sigma-Aldrich) was added to the parasite pellet during PBS wash steps. Pellets were resuspended in 2 $\times$ Reducing Sample Buffer (1% (*w/v*) SDS, 10% (*v/v*) Glycerol, 1 mM EDTA, 50 mM DTT, 0.005% (*w/v*) Bromethyl Blue, 50 mM Tris–HCl pH 6.8) and incubated at 100 °C for 10 min. Lysates were spun at 10,000 $\times$*g* for 5 min. The resulting supernatants were then run on 4–15% precast polyacrylamide gels (Bio-Rad) (150 V, 1–2 h) before being transferred to nitrocellulose membranes using a Transblot Turbo Transfer System (Bio-Rad) according to the manufacturer instructions (25 V, 1.3A, 10 min). Membranes were then blocked in blocking solution (3% (*w/v*) BSA, 2% (*v/v*) Tween-20, in PBS) at RT for 1 h, before being probed with Rabbit-cMyc (Sigma-Aldrich, C3956) and Rabbit-EXP2 antibodies [57], diluted 1:500 in blocking solution at RT for 1 h with agitation. Membranes were then washed three times in PBS-Tween (PBS, 2% (*v/v*) Tween-20) for 5 min. Membranes were then probed using goat α-rabbit horse radish peroxidase (HRP) labelled secondary antibody (Invitrogen, 31,460) diluted 1:10,000 in blocking solution for 45 min with agitation and washed three times in PBS-Tween. Membranes were then incubated in Clarity Western substrate (Bio-Rad) for 1 min at RT and imaged by chemiluminescence on ChemiDoc Imaging System (Bio-Rad).

### Immunofluorescence analysis

Thin blood smears were made of parasite cultures, after which slides were airdried and fixed in a 90% (*v/v*) acetone, 10% (*v/v*) methanol solution, for 2 min. Slides were left to dry overnight, and then used immediately or frozen at – 20 °C. Using a hydrophobic PAP pen (Merck), circles were drawn on slides and allowed to dry for 10 min, and slide were then blocked using 50 μl of blocking solution (5% (*w/v*) BSA, 5% (*v/v*) FBS, 2% (*v/v*) Tween-20, in PBS) and incubated at RT for 1–2 h. Slides were then probed with mouse α-HA antibody (Sigma-Aldrich, 12CA5) at 1:250 dilution, or rat α-HA antibody (Sigma-Aldrich, 3F10) at 1:250 dilution, in blocking buffer at RT for 1–2 h. Antibodies were removed by inverting and washing in PBS-Tween solution for 5 min with agitation, repeating three times. After washing, slides were incubated with either donkey α-mouse (Thermofisher, A-10037) or goat α-rat AlexaFluor 568 secondary antibodies (Thermofisher, A-11077) diluted 1:2000 in blocking solution, incubating at RT in the dark for 1 h. Slides were again washed three times in PBS-Tween, then air dried for ~ 2 min. A single drop of Prolong Antifade (Gold) solution with DAPI (6-diamidino-2-phenylindole) (Invitrogen) was added to each well and a glass coverslip (ProSciTech) applied gently. Slides were incubated in the dark at 37 °C overnight. Slides were imaged on a Nikon Confocal Microscope either immediately or stored at 4 °C, for up to 4 weeks.

### Guanidinium lysis assay

Lysis assays were carried out as previously described [50]. Briefly, parasite cultures were pelleted by centrifugation at 500 $\times$*g* for 5 min and 50 µl aliquots made. To each aliquot, 1 ml of 140 mM guanidinium chloride buffered in 20 mM HEPES (pH 7.4) was added. MT-PBS and 0.15% (*w/v*) saponin were used as negative and positive controls for lysis, respectively. RBCs were gently resuspended and incubated at 37 °C for 5 min. Following incubation, samples were centrifuged at 10,000 $\times$*g* for 1 min and the degree of lysis was determined by measuring the amount of haemoglobin released into the supernatant. 100 µl of supernatant was pipetted into a 96-well plate in triplicate and the absorbance read on spectrophotometer (GloMax Promega) at 560 nm wavelength. Three biological replicates were performed, with results presented as mean $\pm$ standard error of the mean (SEM). The degree of RBC lysis was determined by the optical density (OD) values generated as follows;

$${\text{Degree}}\, {\text{of}}\, {\text{RBC}}\,{\text{ lysis}} \left( \% \right) = \frac{{\left[ {{\text{OD}}\, {\text{solution}} \,{\text{of}}\, {\text{interest}} {-} {\text{OD}}\, {\text{PBS}}} \right]}}{{\left[ {{\text{OD}}\, {\text{saponin}} {-} {\text{OD}}\, {\text{PBS}}} \right]}} \times 100$$

### Phylogenetic analysis

Following manual editing and trimming of the protein sequences, an alignment was created using MUSCLE [58] parameters in Geneious [59]. Phylogenetic reconstruction was conducted in Mega X [60] using the Maximum Likelihood method [61]with 500 bootstraps.

## Results

### Phylogenetic analysis and conservation of the *P. falciparum* and *P. berghei* RhopH complex subunits

**Fig. 1:**
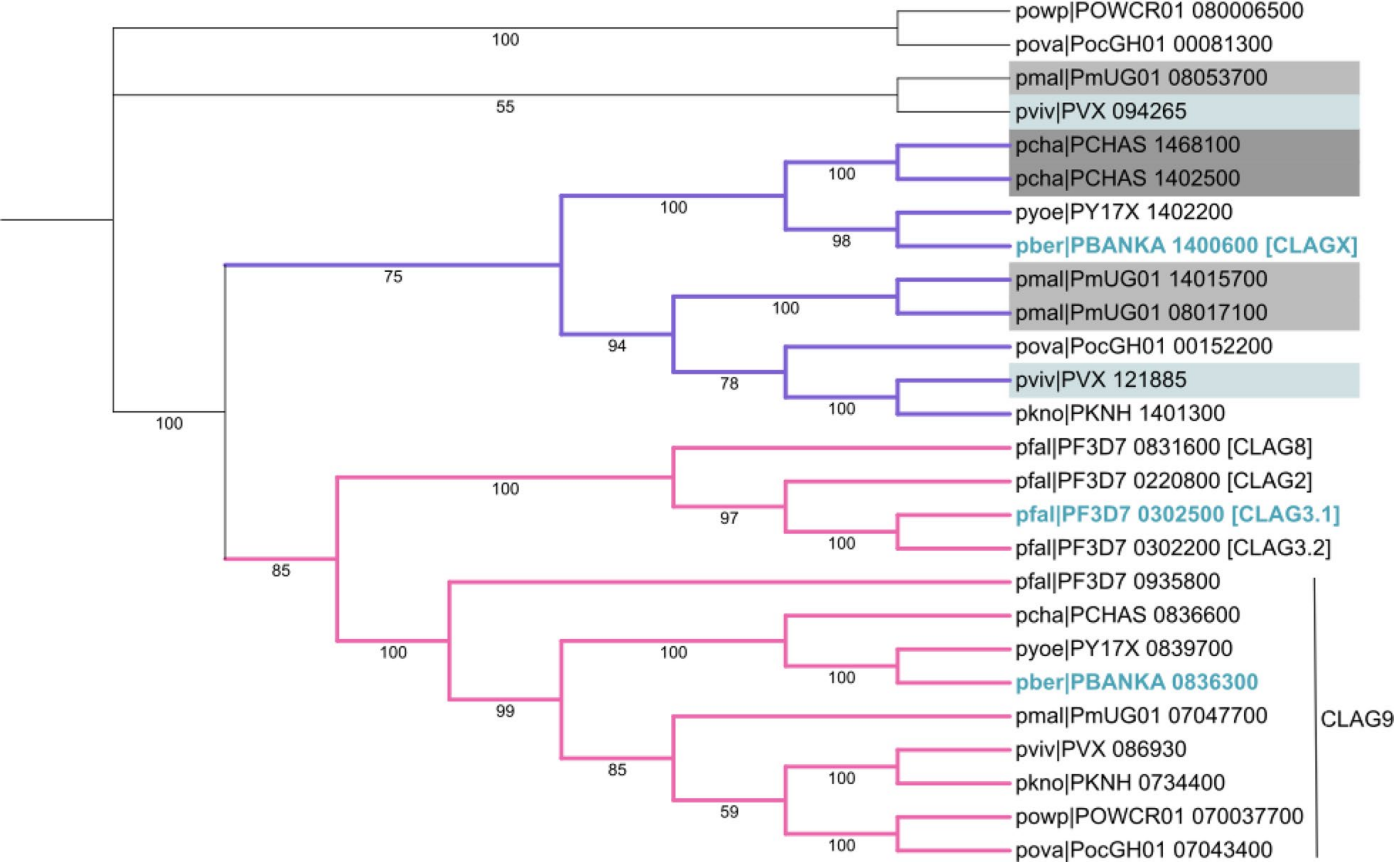
A phylogenetic tree of CLAG protein sequences from human and rodent species. The evolutionary history was inferred by using the Maximum Likelihood method and JTT matrix-based model [64]. Evolutionary analyses were conducted in MEGA X [60]. The bootstrap consensus tree was inferred from 500 replicates [65]. Branches corresponding to partitions reproduced in less than 50% bootstrap replicates are collapsed. The phylogenetic tree was annotated using ITOL[66]. The branches coloured in pink and purple indicate two different clades. The *clag* genes bolded in blue are those analysed in this study. The non-*falciparum* species that contain two or more *clag* genes that are not closely related to *clag9* are highlighted

A previous phylogenetic tree of the *Plasmodium* CLAG proteins was inferred using the Neighbour-Joining method and aligning either only the first or last exon of *P. falciparum, P. vivax* and *P. yoelii clag* genes [29]. Herein, we used the entire CLAG protein sequences from all human *Plasmodium* species and three rodent species, *P. berghei, P. yoelii* and *P. chabaudii,* and constructed a phylogenetic tree using the Maximum Likelihood method (Fig. 1). This revealed that the CLAG proteins fall into at least two distinct clades. One clade (pink branches) comprises the previously annotated Type AP CLAG proteins, including *P. falciparum* (*Pf*) CLAG9, but additionally comprises the Type A *Pf*CLAG proteins (CLAG 2, 3.1, 3.2 and 8). However, *Pf*CLAG9 divergently evolved from the other *Pf*CLAG proteins with the CLAG proteins from other species (also denoted as CLAG9). Notably, all non-*falciparum* species harbour a single CLAG protein in this clade. Another clade (purple branches) contains only CLAG proteins from non-*falciparum* species, and in some cases, there are multiple CLAG proteins for each species.

**Fig. 2:**
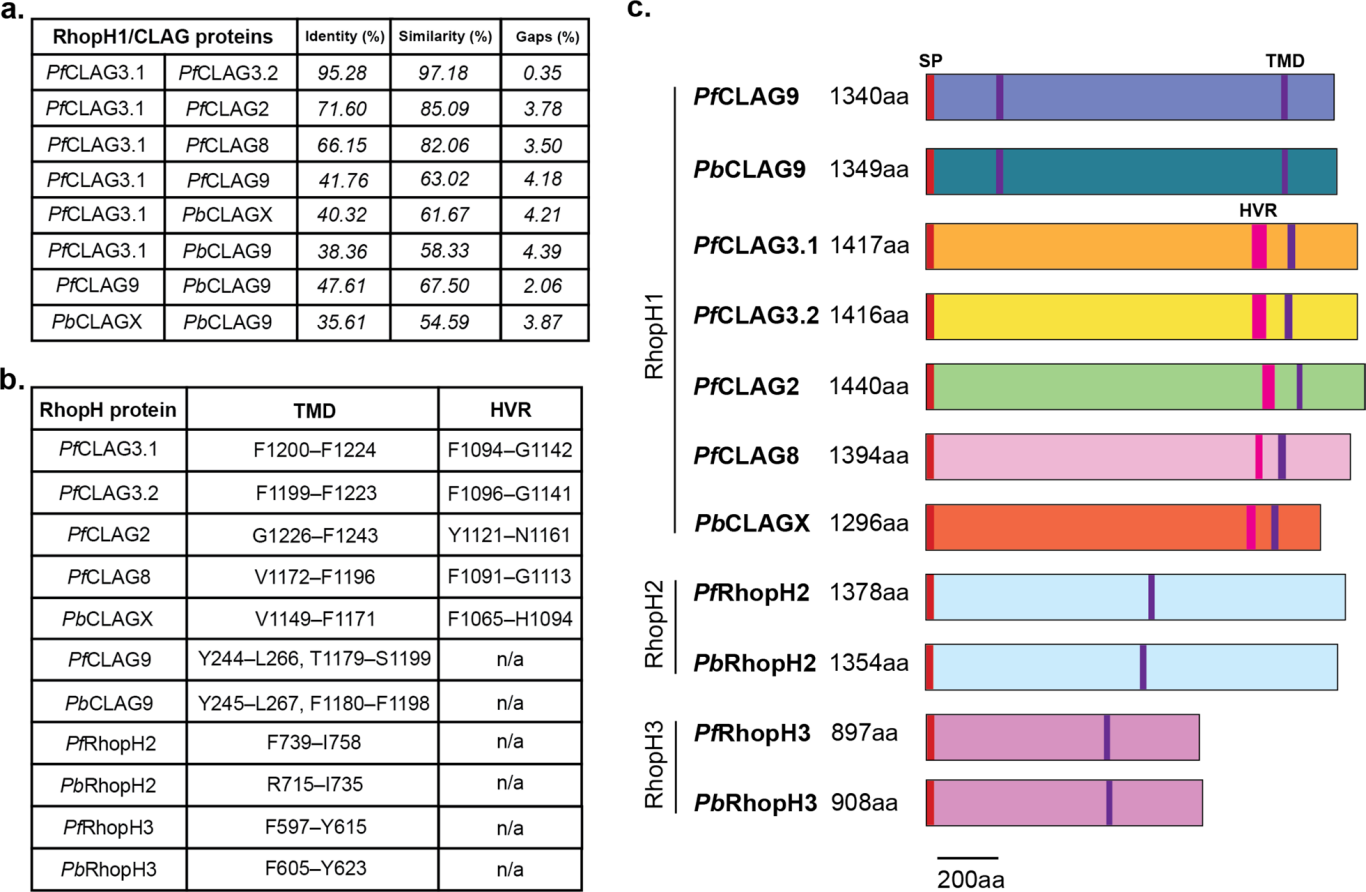
Comparison of *P. falciparum* and *P. berghei* RhopH complex proteins. **a** Identity, similarity and gaps shared between RhopH1/CLAG proteins in both *P. falciparum* and *P. berghei*. **b** The location of transmembrane domains (TMD) was derived from consensus between InterPro [67], SMART [68], DeepTMHMM [69], and TOPCONS [70] TMD prediction software. Hypervariable regions (HVR) are indicated. **c** Alignment of RhopH complex proteins derived from *P. falciparum* and *P. berghei* set to scale. SP, signal peptide

A potential indicator for the ability of CLAG proteins to functionally complement one another is conservation of protein sequences as well as functional domains. Figure 2a shows the percentage amino acid identity and similarity shared between each CLAG component within *P. falciparum* (*Pf*) and between *P. falciparum* and *P. berghei* (*Pb*). *Pf*CLAG3.1 and *Pf*CLAG3.2, show a high degree of conservation (97% similarity), which is upheld, although to a lesser degree, with *Pf*CLAG2 and *Pf*CLAG8, with similarity values of 85% and 82%, respectively. *Pf*CLAG3.1 shows the lowest similarity to *Pf*CLAG9 (63% similarity) (Fig. 2a), which possibly denotes a divergent function. Protein similarities between *Pf*CLAG3.1 and *Pb*CLAGX are less conserved (Fig. 2a), [62, 63]. Yet, as outlined further below, the regions such as the predicted TMDs, HVRs and RhopH2/RhopH3 binding regions are maintained between these two CLAG proteins (Fig. 2b, c and Figure S1). The same can be said for *Pf*CLAG9 and *Pb*CLAG9 (Fig. 2). Conversely, *Pb*CLAGX and *Pb*CLAG9 encoded proteins are the least conserved, also hinting at an independent function of the two proteins. Similarly, conservation of *Pf*CLAG3.1 and *Pb*CLAG9 is not strongly upheld (Fig. 2a).

Analysis of the presence and position of TMDs in *Pf*RhopH and *Pb*RhopH subunits was assessed utilising InterPro [67], SMART [68], DeepTMHMM [69] and TOPCONS [70] (Fig. 2b, c). *P. falciparum* and *P. berghei* CLAG9 proteins were confidently predicted by all four software to contain two TMDs*.* Conversely, *Pf*CLAG2, 3.1, 3.2 and 8 as well as *Pb*CLAGX were consistently and confidently predicted to have a single TMD within the C-terminal (Ct) region (Fig. 2b, c), although several other TMDs were less confidently predicted and by not all programs. *Pf*CLAG3, *Pf*RhopH2 and *Pf*RhopH3 single TMDs have been experimentally validated in the literature [71–73]. Some dissimilarities were observed regarding the number of TMDs present in *Pf*CLAG9*,* with up to four described [44], and additional putative TMDs in the other *Pf*CLAG proteins have been reported, depending on the prediction software utilised [18, 27].

The HVR has been shown previously to be restricted to *Pf*CLAG2, 3.1, 3.2 and 8 proteins but is not observed in *Pf*CLAG9 (Fig. 2b, c) [73, 74]. The HVR comprises a surface exposed extracellular loop, which has in part contributed to the *Pf*CLAG3 proteins suggested role as the channel or pore component of the NPPs in *P. falciparum* [18, 33]. The lack of a HVR in *Pf*CLAG9 indicates it is not exposed at the surface of the RBC membrane [22]. Thus taken altogether, the *Pf*CLAG proteins have gone through a separate evolutionary change to *Pb*CLAGX and belong to a different clade, yet the phylogenetic analysis and the conservation in number and presence of TMDs and HVRs indicate *Pb*CLAGX is more closely related to the *Pf*CLAG proteins than to *Pb*CLAG9*,* and that *Pb*CLAG9 is more closely related to *Pf*CLAG9. From this we hypothesise that *Pb*CLAGX and *Pb*CLAG9 have different functions and that the functional ortholog of *P. falciparum clag3* is *PbclagX.*

Finally, the structure of *P. falciparum* soluble RhopH complex has revealed detailed regions of CLAG3.1 that bind RhopH2 and RhopH3, and thus are likely essential to complex formation [71]. These binding regions are also present in *Pb*CLAGX and *Pb*CLAG9 (Figure S1)*.* These observations are in keeping with the maintenance of this complex and the NPPs across the two species and support the utility of *P. berghei* as a model to study CLAG function and essentiality.

### *Plasmodium berghei clagX and clag9 *cannot be knocked out individually in *P. berghei*

To first evaluate the essentiality of *clag* genes in the rodent malaria model, a conventional knockout of *Pbclag9* and *PbclagX* was individually attempted, utilising plasmid constructs designed by the Sanger Institute *Plasmodium* Genetic Modification project (*Plasmo*GEM) [75]. The two constructs PBGEM-256188 (*clagX*) and PBGEM-260364 (*clag9*) were designed to facilitate double cross over homologous recombination, to replace each gene with the hDHFR drug selectable marker that had been C-terminally tagged with 3 $\times$ HA epitope (Figure S2 and Figure S3). Transfections of *P. berghei* with each construct were performed three times, but parasites were either never recovered after drug selection or returned parasites were not positive for integration, alluding to both genes being essential for parasite survival (Figure S2 and Figure S3). This suggests the absence of any functional redundancy between *Pb*CLAGX and *Pb*CLAG9 and thus to examine this further, we attempted a functional replacement of *Pb*CLAGX.

### Examining complementation and functional conservation of CLAG proteins to design functional replacements

To assess whether *Pb*CLAG9 can functionally complement *Pb*CLAGX and if the latter is the functional ortholog of *Pf*CLAG3.1, a strategy to replace the sequences encoding *Pb*CLAGX with that of *Pb*CLAG9 or *Pf*CLAG3.1 was devised. As previously illustrated, the TMDs, HVRs and RhopH2/RhopH3 binding regions are likely to be essential to CLAG function. Given that the allelic replacement of the entire *PbclagX* gene, over 3.8 kb in size, would be technically more difficult, the functional regions lying within the C-terminal (Ct) region were replaced.

To determine the appropriate regions of *Pb*CLAGX to replace, multiple sequence alignments between *Pb*CLAG9 (*Pb*ANKA_0836300), *Pb*CLAGX (*Pb*ANKA_1400600), and *Pf*CLAG3.1 (PF3D7_0302500) (Figure S1) were analysed. Based on the structure of the soluble RhopH complex, *Pf*CLAG3.1 contains three binding regions with RhopH2, all sitting centrally in the ‘RhopH2 bridge’ [71, 73]. Another two binding regions within *Pf*CLAG3.1 bind to RhopH3, one in the N-terminus (Nt) and the other in the C-terminus (Ct), however, the Nt region was considered too far upstream for replacement. Each of these binding regions are very well conserved across *Pb*CLAGX*, Pb*CLAG9 and *Pf*CLAG3.1 (Figure S1). Specifically, of the 17 residues described by Schureck, Darling [71] considered to be important for the binding of *Pf*CLAG3.1 and RhopH2, 10 are identical, 6 are conserved amino acids, and only 1 aa is not conserved (Figure S1). In the Ct RhopH3 binding site, of the 8 essential amino acids that stabilize the complex, 6 are identical, 1 is conserved and 1 is partially conserved (Figure S1).

*Pb*CLAGX and *Pb*CLAG9 are the least conserved in the Ct region and in the region that extends slightly further upstream. The region of *Pb*CLAG9 to replace *Pb*CLAGX was consequently chosen from S888 to encompass the Ct bundle, including a single RhopH2 binding region, *Pb*CLAGX TMD and HVR, and Ct RhopH3 binding region (Figure S1). When comparing *Pf*CLAG3.1 and *Pb*CLAGX, apart from the signal sequence and a small proportion of the sequence following this, the sequences are relatively well conserved until just upstream of the HVR, after which the sequences become more divergent. Therefore, we decided to replace *Pb*CLAGX from S1060, with the corresponding aligned *Pf*CLAG3.1 sequence (Figure S1), as this region contains the predicted HVR and TMD of both sequences and the Ct RhopH3 binding region. An additional allelic replacement of *Pb*CLAGX with *Pf*CLAG3.1 utilised sequences even further upstream, beginning at K654 to further encompass all RhopH2 binding regions (Figure S1).

### Replacement of *P. berghei clagX* with *P. berghei clag9*

**Fig. 3:**
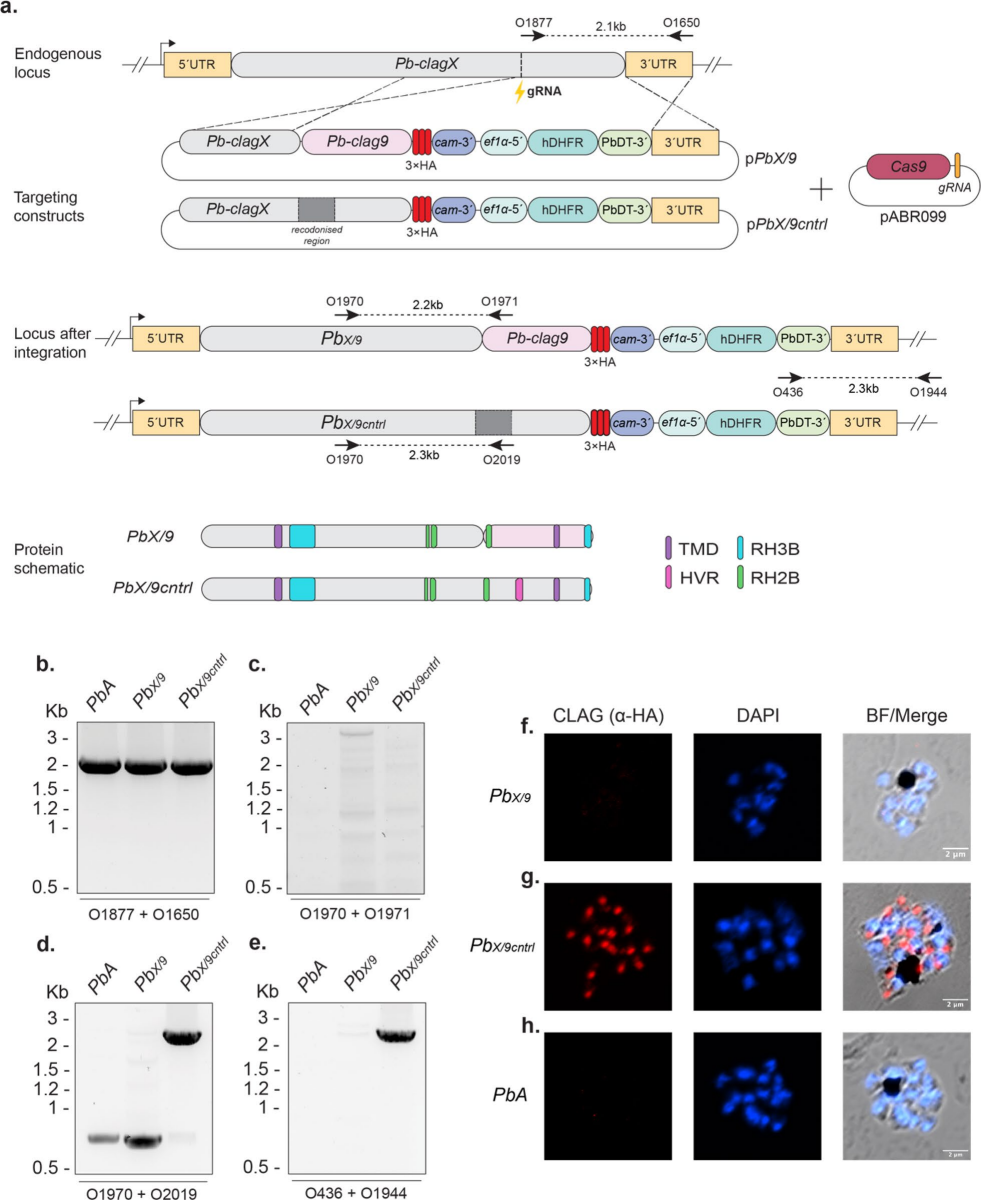
*P. berghei clag9* cannot complement the function of *clagX*. **a** Schematic of p*PbX/9* designed to replace the ~ 450 aa Ct portion of the *PbA clagX* locus with that of the *clag9* locus (pink). A p*PbX/9cntrl* construct was used as a control for transfection. pABR099 containing a *PbclagX* gRNA was utilised to cleave the *PbclagX* locus (indicated by lightning bolt). A schematic of the expected *PbclagX/9* (*Pb*_*X/9*_) or *PbclagX/9control* (*Pb*_*X/9cntrl*_) locus after integration (int) of the targeting construct is shown, as is a protein schematic to highlight which domains have been included in the replacement. TMD, transmembrane domain; RH2B, RhopH2 binding site; RH3, RhopH3 binding site; HVR, hypervariable region. Indicated are the primers used to validate the presence of **b** WT; **c** 5´Int of *Pb*_*X/9*_; **d** 5´Int in *Pb*_*X/9cntrl*_; and **e** 3´Int of *Pb*_*X/9*_ and *Pb*_*X/9cntrl*_*,* and the expected product sizes are indicated. *PbA* gDNA was used as a positive control for detecting WT locus and as a negative control for an integrated locus. **g**–**i** IFA of schizont stage parasites from the indicated lines were probed with α-HA antibodies. Successful allelic replacement of the *PbclagX* gene should lead to expression of the HA epitopes. Brightfield image merged with DAPI and α-HA shows; **g** negative HA expression in *Pb*_*X/9*_; and **h** positive HA expression localising to what appears to be the rhoptries in *PbX/9cntrl* parasites. **i** *PbA* parasites used as a negative HA expression control

The plasmid construct p*PbclagX/Pbclag9*-HA (p*PbX/9*) was designed to target the *PbclagX* locus and replace it with the corresponding *Pbclag9* region, which included the HVR and TMD located in the Ct region (Figure S1 and Fig. 3a), in frame and incorporating 3 $\times$ HA epitope tags. Transfections were performed in combination with the CRISPR-Cas9 plasmid (pABR099) incorporating a gRNA that facilitated targeted gene repair/editing. In conjunction with the p*PbX/9* allelic replacement, a p*PbclagX/9control*-HA (p*PbX/9cntrl*) construct was designed to replace the target region with recodonised *PbclagX* sequence. Thus, p*PbX/9cntrl* functioned as a control for transfection and for the possibility that addition of the HA tags could have a detrimental impact on the folding and/or functionality of the encoded protein.

Transfections of *P. berghei* ANKA (*PbA*) parasites were carried out on four separate occasions and PCR results on recovered gDNA are representative of all transfection outcomes (Fig. 3b–d). On each instance parasites were recovered, usually between 7 and 14 days after transfection and drug selection. Each transfection returned a positive PCR result for wildtype (WT) parasites transfected with both p*PbX/9* and p*PbX/9cntrl* (Fig. 3b), indicating episomal expression of either the targeting or Cas9 plasmid conferring drug resistance. At no point did parasite populations transfected with p*PbX/9* return a positive PCR result for 5´integration (5´Int) with O1970/O1971 oligonucleotide combination, despite optimisations of primers and PCR conditions (Fig. 3c). Conversely, parasites transfected with p*PbX/9cntrl* returned positive integration results by PCR with O1970/O2019 (Fig. 3d). Additionally, 3´integration (3´Int) primers (O436/O1944) confirmed generation of the *Pb*_*X/9cntrl*_ line and the negative establishment of *Pb*_*X/9*_ (Fig. 3e). Immunofluorescent analysis (IFA) of *Pb*_*X/9cntrl*_ transgenic parasites using α-HA antibodies also confirmed the successful tagging of parasites, and the absence of an epitope tag in recovered parasites transfected with p*PbX/9* (Fig. 3f–h).

Given the control construct was successfully able to modify the locus by the addition of epitope tags and that parasites transfected with p*PbX/9* did not return positive results for integration by PCR on four separate occasions, we conclude that the allelic replacement of *PbclagX* Ct region could not be complemented with the corresponding region of *Pbclag9*. It would also suggest that the endogenous *Pbclag9* gene was unable to compensate for the loss of *PbclagX,* supported by our preliminary work (Figure S2)*.* From these results we infer that the *clag* genes in *P. berghei* are unable to complement the function of one another.

### Replacement of *P. berghei clagX* with critical domains of *P. falciparum clag3.1*

**Fig. 4:**
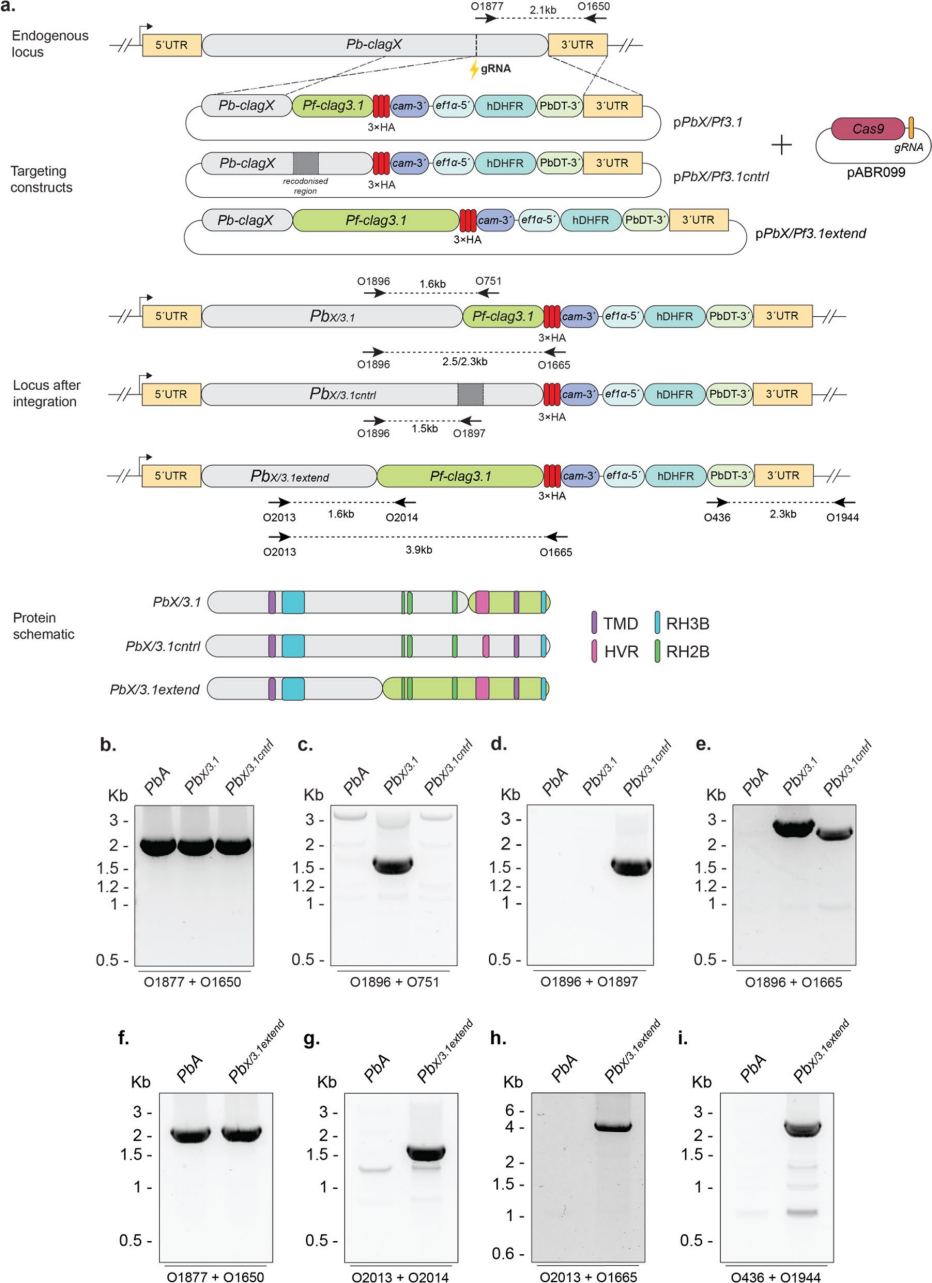
*P. berghei clagX* can be complemented by* P. falciparum clag3.1*. **a** Schematic of p*PbX/Pf3.1* and p*PbX/Pf3.1extend* designed to replace either the ~ 350 aa or ~ 730 aa Ct portion of the *PbA clagX* locus, respectively, with that of the *Pfclag3.1* locus (green). The p*PbX/Pf3.1cntrl* construct was used as a control for transfection. pABR099 containing a *PbclagX* gRNA was utilised to cleave the *PbclagX* locus (lightning bolt). A schematic of the expected *PbclagX/Pfclag3.1* (*Pb*_*X/3.1*_), *PbclagX/Pfclag3.1control* (*Pb*_*X/3.1cntrl*_) or *PbclagX/Pfclag3.1extended* (*Pb*_*X/3.1extend*_) locus after integration of the targeting construct is shown, as is a protein schematic to highlight which domains have been included in the replacement. TMD, transmembrane domain; RH2B, RhopH2 binding site; RH3, RhopH3 binding site; HVR, hypervariable region. Indicated are the primers used to validate the presence of **b** WT; **c** *Pb*_*X/3.1*_ int; **d** *Pb*_*X/3.1cntrl*_ int; **e** HA tag int in *Pb*_*X/3.1*_ and *Pb*_*X/3.1cntrl*_; **f** *Pb*_*X/3.1extend*_ WT; **g**–**i** and *Pb*_*X/3.1extnd*_ int, with the respective product sizes indicated. *PbA* gDNA was used as a positive control for detecting WT locus and as a negative control for an integrated locus

We next aimed to determine whether *clagX* is the functional ortholog of *P. falciparum clag3*. To demonstrate this, the plasmid construct p*PbclagX/Pfclag3.1*-HA (p*PbX/Pf3.1*) was designed to replace the Ct region of *PbclagX,* which contains the predicted TMDs and HVR with *Pfclag3.1* (Figure S1 and Fig. 4a)*.* Another plasmid construct p*PbclagX/Pfclag3.1control*-HA (p*PbX/Pf3.1cntrl*) was designed that contained a recodonised *PbclagX* sequence in place of the region of replacement, to be used as a control for transfection (Fig. 4a).

Transfection of *PbA* parasites with the two targeting constructs were conducted and parasites were recovered in each instance within 7–14 days. Drug resistant populations of parasites were positive for WT in each instance (Fig. 4b), indicating episomal expression of either plasmid construct. Recovered parasites positive for integration with O1896/O751 primers indicated successful generation of the *Pb*_*X/3.1*_ line (Fig. 4c), and the *Pb*_*X/3.1cntrl*_ line was also positive for integration with O1896/O1897 primers (Fig. 4d). In addition, a reverse primer unique to the 3 $\times$ HA tag (O1665) was also used to confirm successful generation of the *Pb*_*X/3.1*_ and *Pb*_*X/3.1cntrl*_ parasites (Fig. 4e).

**Fig. 5:**
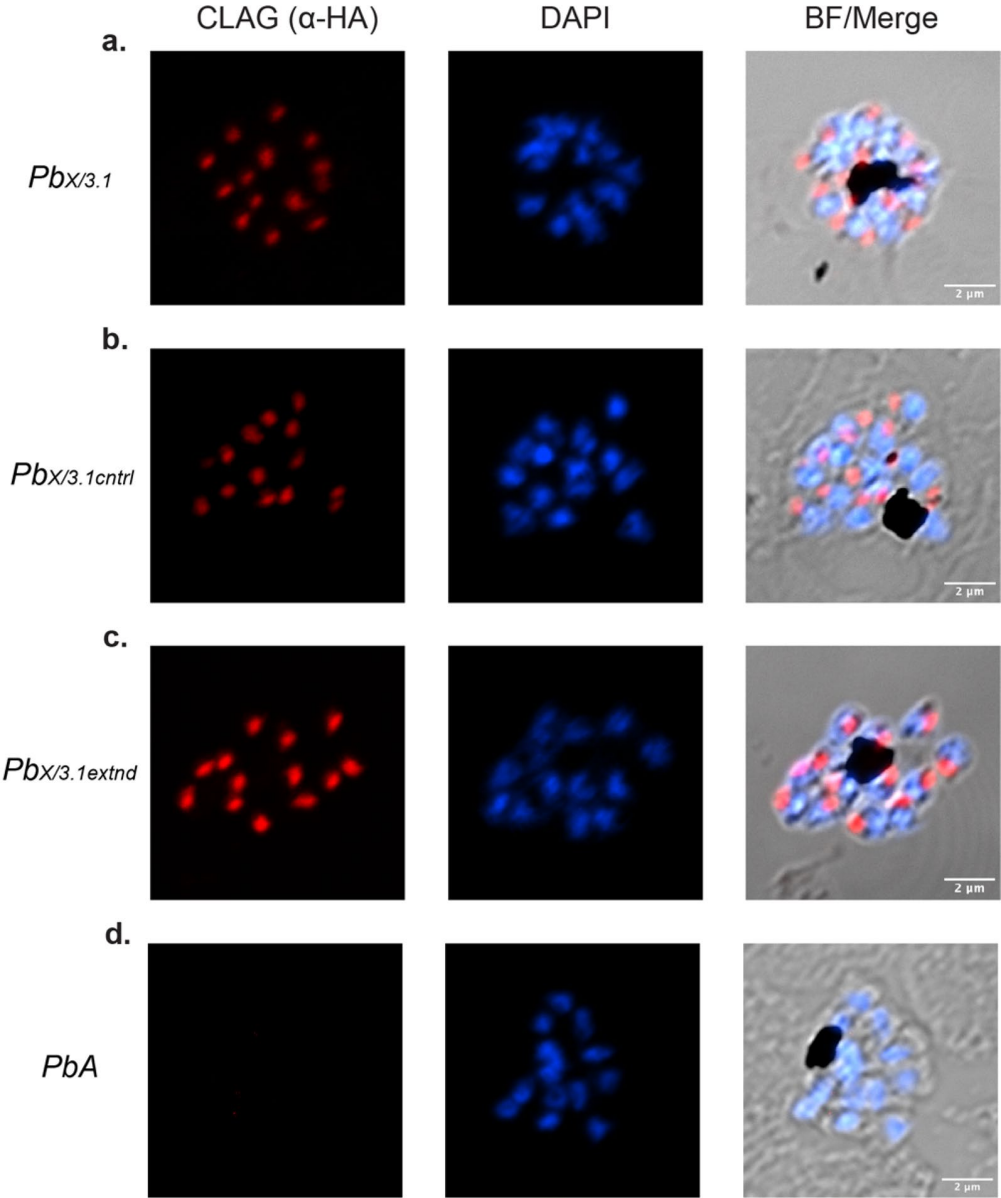
Immunofluorescent analysis of tagged *Pb*_*X/3.1*_*, Pb*_*X/3.1cntrl*_ and *Pb*_*X/3.1extnd*_ lines confirm *PbclagX* can be replaced with *Pfclag3.1*. Schizont stage parasites from the indicated lines were probed with α-HA antibodies. Successful allelic replacement of the *PbclagX* gene should lead to expression of the HA epitopes. Brightfield image merged with DAPI and α-HA shows HA expression localising to what appears to be the rhoptries after replacement of **a** *Pb*_*X/Pf3.1*_*,* **b** *Pb*_*X/3.1cntrl*_*,* **c** and *Pb*_*X/3.1extend*_. **d** *PbA* was used as a negative HA expression control

To be confident that the *PbclagX* region chosen for replacement by *Pfclag3.1* was large enough to convey species functionality, an extended allelic replacement construct was designed. This targeting construct, p*PbclagX/Pf3.1extend*-HA (p*PbX/Pf3.1extend*), was identical to p*PbX/Pf3.1* with the exception that it included *Pfclag3.1* sequence encoding ~ 730 aa, which is more than twice the replacement size of *PbX/Pf3.1* (Fig. 4a). This *Pf*CLAG3.1 sequence encompasses all three of the RhopH2 binding regions in the RhopH2 bridge, hypothesised to be critical for RhopH complex formation (Fig. 2). Following transfections of *PbA* with p*PbX/3.1extend,* parasite populations were positive for WT by PCR using O1877/O1650 primers (Fig. 4f), indicative of episomal plasmid expression. PCRs of transgenic parasites using 5´ and 3´ integration specific primers O2013/O2014, O2013/O1665 and O426/O1944 confirmed generation of the *Pb*_*X/3.1extend*_ line and successful tagging with HA epitope tag (Fig. 4g–i). IFA of transgenic parasites using α-HA antibodies validated the successful tagging of *Pb*_*X/Pf3.1*_, *Pb*_*X/3.1cntrl*_ and *Pb*_*X/Pf3.1extend*_ parasites. Punctate labelling of the hybrid CLAGX/3.1 protein was observed at the apical end of merozoites within a schizont, indicative of rhoptry localisation although this was not validated (Fig. 5).

It should be noted that the extended *Pfclag3.1* region lies further upstream than the corresponding region of *Pbclag9* used in the p*PbX/9* construct*.* The combined failure to knockout *PbclagX* and to replace *PbclagX* with *Pbclag9,* but the successful allelic replacement of *PbclagX* with *Pfclag3.1* indicates the latter are functional orthologs, and that the relationship between the *Pb*CLAG proteins is not one of functional redundancy.

### Inducible knockout of *P. berghei clagX* demonstrates essentiality for parasite survival and NPP functionality

**Fig. 6:**
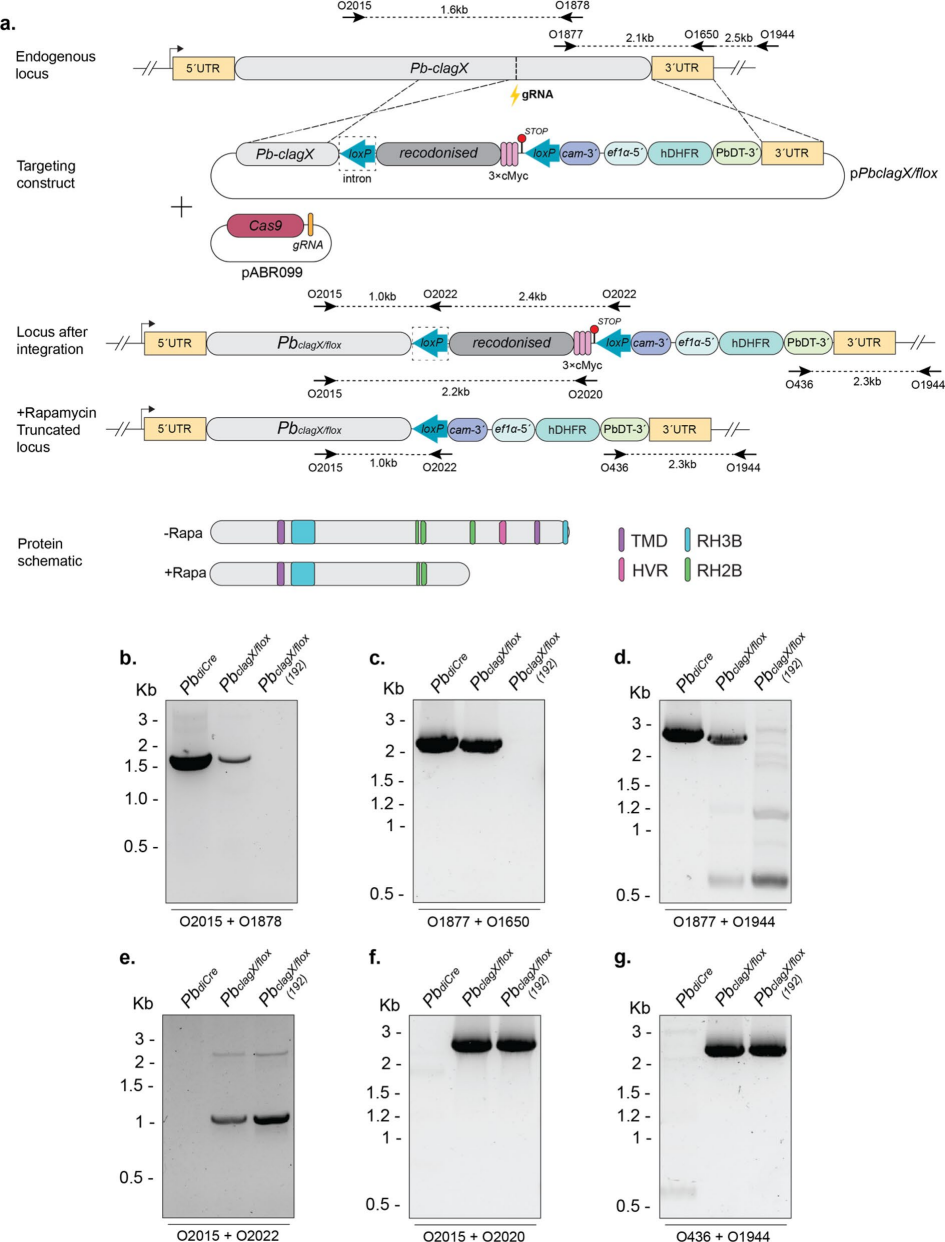
Creation of the *PbclagX/loxP* inducible knockout line. **a** Schematic of p*PbclagX/flox* designed to modify the *Pb*_*diCre*_* clagX* locus such that the ~ 400 aa region containing RhopH2/RhopH3 binding regions (RH2B, RH3B), the hypervariable region (HVR) and transmembrane domain (TMD) are *floxed*. pABR099 containing a *PbclagX* gRNA was utilised to cleave the *PbclagX* locus (lightning bolt). A schematic of the expected *Pb*_*clagX/flox*_ locus after integration of the targeting construct, and the expected truncated locus following the addition of rapamycin and *loxP* recombination, as is a protein schematic to highlight which domains have been lost after truncation. *Pb*_*clagX/flox(192)*_ is a clonal line of *Pb*_*clagX/flox*_ generated by serial dilution. Indicated are the primers used to validate the presence of **b**–**d** WT; **e** integration of *loxP*; **f** presence of cMyc; **g** and 3′ integration, and the expected product sizes are indicated. *Pb*_*diCre*_ gDNA was used as a positive control for detecting WT and as a negative control for integration

The failure to knockout the *PbclagX* gene (Figure S2) or to replace it with *Pbclag9* (Fig. 3)*,* indicates this gene is likely to be essential for in vivo growth and survival of parasites. To provide definite proof, a conditional *PbclagX* knockout was created utilising the diCre-*loxP* system [53]. The plasmid construct p*Pb*(diCre)*clagX/loxP*-cMyc (p*PbclagX/flox*) (Fig. 6a) was assembled so that the Ct region (~ 400 aa) of the endogenous *PbclagX* locus, beginning at S896, was flanked with *loxP* sites (Fig. 2). This placed the 5*´ loxP* site in the intron between exons 7 and 8, which encompasses the predicted HVR, TMD and RhopH complex binding regions. The *PbclagX flox* region was recodonised to force recombination upstream of the first *loxP* site and incorporated an in-frame 3 $\times$ cMyc epitope tag followed by a 3*´ loxP* site (Fig. 6a).

The p*PbclagX/flox* construct was transfected into the *Pb*_*diCre*_ marker free line, which expresses Cre59 and Cre60 fused to the FK506-rapamycin-binding protein (FKBP) and FKBP-rapamycin binding (FRB) kinase domain, respectively, in the *p230p* locus under the bi-directional *ef1α* promotor. Transfectants were PCR positive for both WT using O2015/O1878, O1877/O1650 and O1877/O1944 primers (Fig. 6a–c), and for 5*´* and 3*´* Int using O2015/O2022, O2015/O2020 and O436/O1944 primers, indicating parasites in the population comprised *Pb*_*clagX/flox*_. Importantly, both *loxP* sites were integrated (Fig. 6e), and the 3 $\times$ cMyc epitope tag was present (Fig. 6f). To remove WT parasites and obtain a clonal *Pb*_*clagX/flox*_ line, the population was cloned out by limiting dilution. The recovered *Pb*_*clagX/flox(192)*_ line was confirmed to be clonal by PCR, as no amplification product was obtained when using primers capable of detecting a WT locus, whereas the correct size amplification products were obtained for primers that detect an *Pb*_*clagX/flox*_ integrated locus (Fig. 6b–g).

**Fig. 7:**
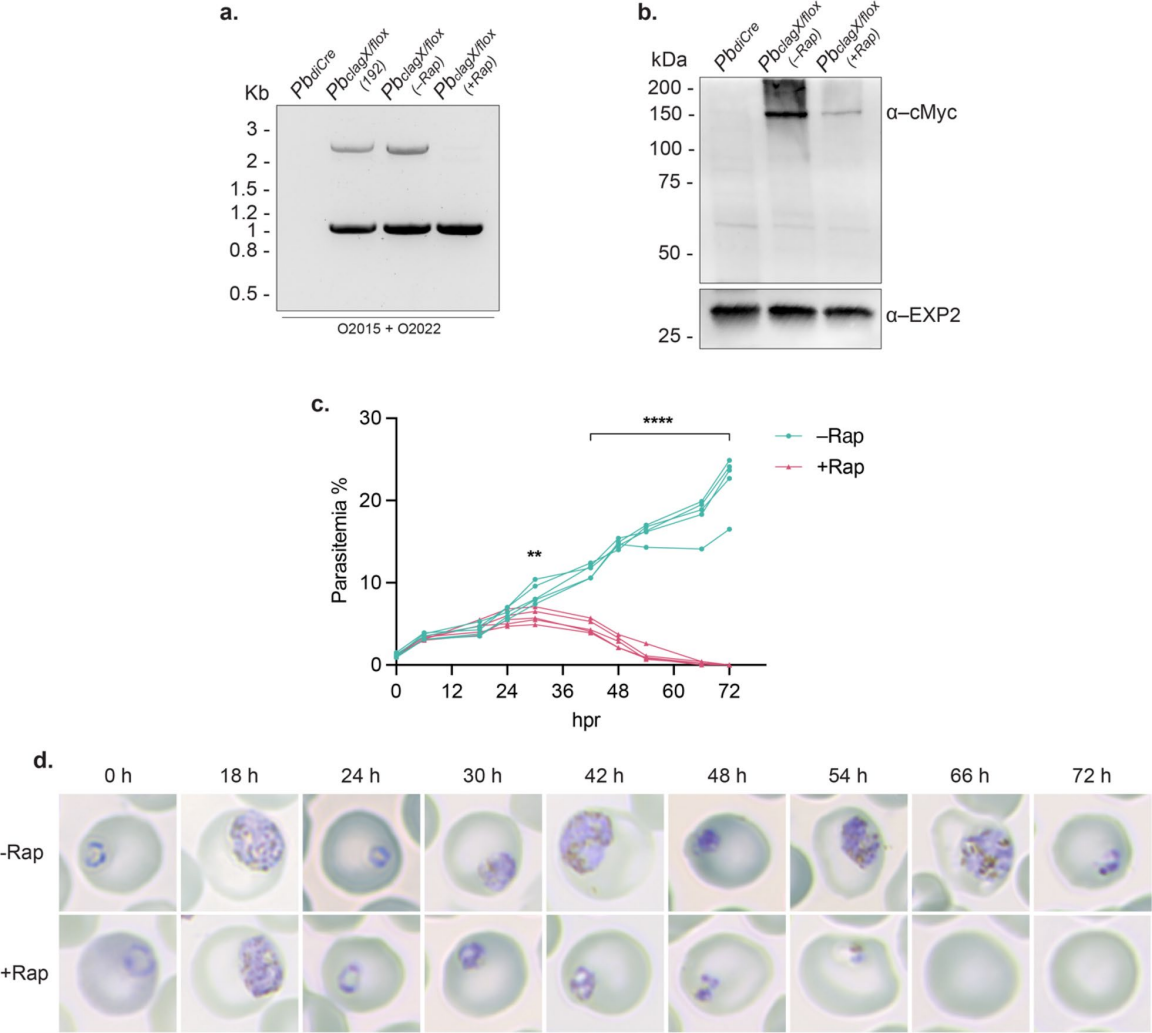
*P. berghei* CLAGX is essential to in vivo parasite growth. **a** PCR used to confirm the recombination of *loxP* sequences that *flox* the *PbclagX* Ct region, after treatment with rapamycin. *Pb*_*clagX/loxP*_ were either grown with vehicle control (–Rap) or with 200 nM rapamycin (+ Rap). A 2.2 and a 1.0 kb band was amplified in *Pb*_*clagX/flox*_ (–Rap) as expected, whereas only a 1.0 kb band was amplified in *Pb*_*clagX/flox*_ (+ Rap), confirming *loxP* recombination. *Pb*_*diCre*_ and *Pb*_*clagX/flox(192)*_ parent line gDNA was used as negative and positive controls, respectively. **b** Western-blot showing the expected ~ 150 kDa cMyc tagged *Pb*CLAGX product in the –Rap schizonts, which is less detectable after parasites are cultured with + Rap. EXP2 serves as the loading control. **c** Parasitemia of mice (n = 5) administered with vehicle control (–Rap) or rapamycin (+ Rap). The timeline reflecting hours post rapamycin-induction (hpr). Statistical significance determined using a two-way ANOVA comparing –Rap and + Rap at each timepoint, followed by Sidak’s multiple comparisons test (**p ≤ 0.01, ****p ≤ 0.0001). **d** Giemsa-stained smears of tail blood from –Rap and + Rap treated mice were used to determine the stage of parasites in (**c**)

Following the production of a clonal *PbclagX/flox* line, the induction of *PbclagX* truncation was performed. For this, synchronous ring stage *Pb*_*clagX/flox*_ parasites were harvested from infected mice and cultured in vitro to schizonts in the presence of 200 nM rapamycin. The excision of the *PbclagX flox* region was confirmed by PCR analysis of gDNA using primer combinations that detect successful recombination of the *loxP* sites (Fig. 6a and Fig. 7a). The excision of the cMyc epitope tags after rapamycin induction was also confirmed by analysing schizont parasite extract by western blot, where 9.8% (± 4.3) *Pb*CLAGX protein remained in + Rap cultures within the single cycle, relative to an α-EXP2 loading control (Fig. 7b and Figure S4).

Next, we assessed the essentiality of *Pb*CLAGX to the in vivo growth of parasites. For these experiments, mice were injected IV with purified *Pb*_*clagX/flox*_ schizonts to achieve a synchronous population of in vivo parasites. The recombination of the *loxP* sites was induced by IP injection of 4 mg/kg rapamycin ~ 24 h post infection when parasites had re-invaded RBCs. An equivalent number of infected mice were instead injected with the vehicle control (DMSO diluted in PBS) as a non-induced negative control. The parasitemia in mice and the health and morphology of parasites was then monitored by Giemsa smears at 6–12 h intervals for 72 h, representing approximately four lifecycles. The parasitemia of induced parasites (+ Rap) was significantly different from the control (–Rap) at ~ 30 h following rapamycin injection (Fig. 7c, d and Figure S5). This corresponds to when parasites should be entering early trophozoite stages of growth in the cycle following rapamycin injection according to the stage of parasites in vehicle control-treated (–Rap) mice (Fig. 7d and Figure S5). Hereafter, the parasitemia in + Rap treated mice decreased steadily, with parasites unable to progress past the second cycle of growth. By 66–72 h, all parasites had died and/or had been cleared from circulation, compared to the parasites in control-treated (–Rap) mice, which were entering their fourth cycle of growth (Fig. 7c, d and Figure S5). These observations confirm the essentiality of *Pb*CLAGX to the parasite lifecycle and provide the first functional evidence for CLAG essentiality to *Plasmodium* parasite survival in vivo.

**Fig. 8:**
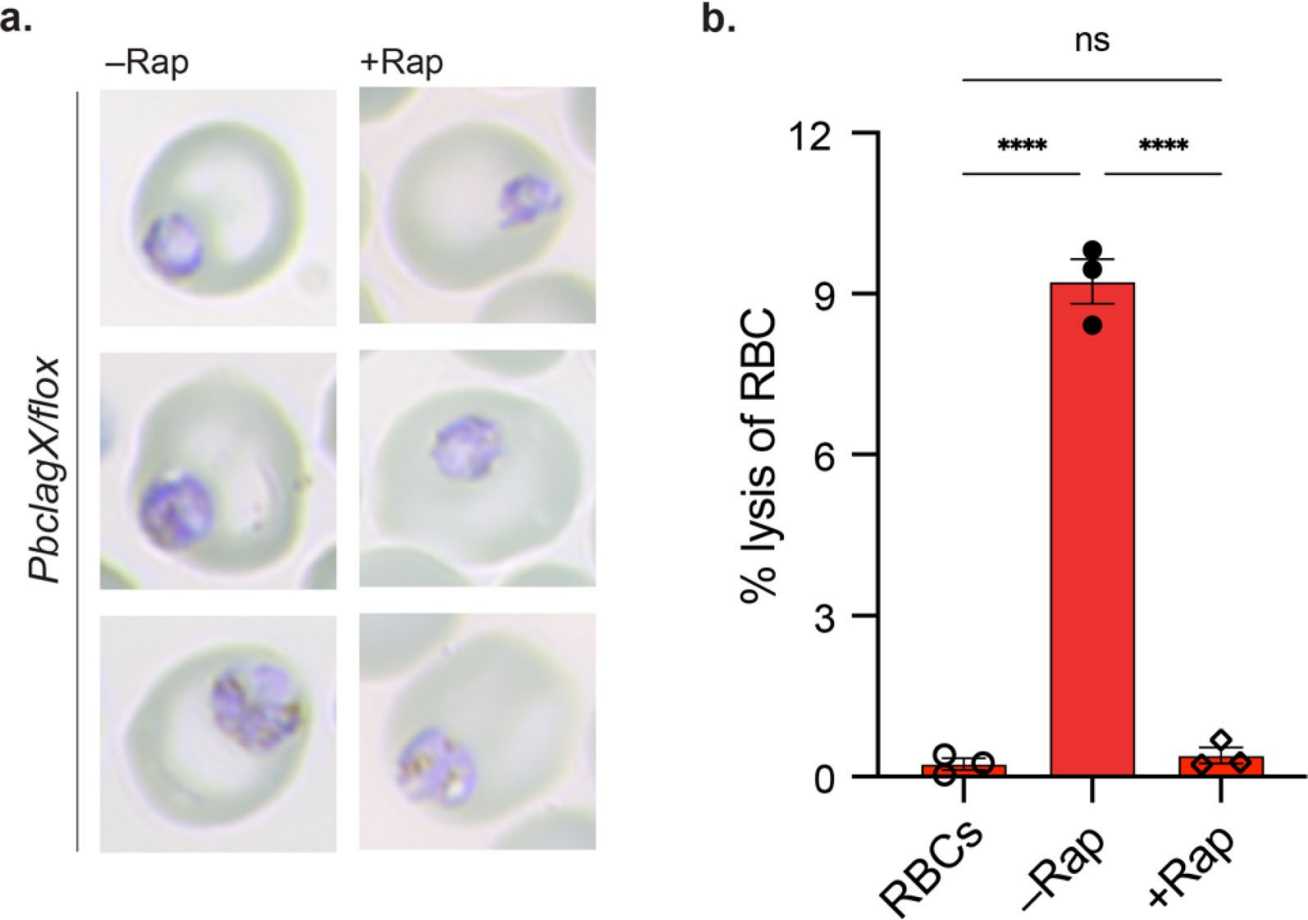
*PbclagX/flox* parasites induced for *clagX* excision have perturbed NPP activity. **a** Giemsa images of infected RBCs harvested from mice infected with *PbclagX/flox* parasites that were administered either vehicle control (–Rap) or 4 mg/kg rapamycin (+ Rap), at ~ 30 hpr (n = 3 per group). **b** Parasitised RBCs (~ 10% parasitemia) harvested from –Rap and + Rap mice and uninfected RBCs (negative control) were resuspended in guanidium chloride (140 mM) lysis solution and the percentage of RBC lysis measured by haemoglobin absorbance, calculated relative to lysis with saponin (100% lysis) (n = 3). Statistical significance determined using a one-way ANOVA followed by Tukey’s multiple comparisons test (no significance (ns), ****p ≤ 0.0001)

To determine if there was a negative impact to NPP functionality following the knockout of *Pb*CLAGX, a guanidinium chloride-induced haemolysis assay was used on in vivo rapamycin induced *PbclagX/flox* parasites. The assay has been shown in our previous publication to rapidly induce lysis of parasitised RBCs, specifically via NPP uptake of guanidinium chloride [50]. *PbclagX/loxP* infected mice were administered with either vehicle control (–Rap) or 4 mg/kg rapamycin (+ Rap) as described for Fig. 7, however, this time parasites were harvested from mice prior to the ~ 30 hpr timepoint as following this point the + Rap and –Rap groups started to demonstrate differences in growth (Fig. 7c, d). At this timepoint parasites were at early trophozoite stage (Fig. 8a and Figure S6) and would normally have functional NPPs [50]. Pellets from uninfected RBCs (negative control) and iRBCs at 10% parasitemia (+ Rap, − Rap) were subjected to the guanidinium lysis assay, and the lysis was measured by the release of haemoglobin. Uninfected RBCs had an average lysis value of 0.23% (± 0.19) in comparison to the significantly higher 9.23% (± 0.73) lysis of the non-induced (–Rap) group, indicating that the majority of the parasites were presenting with functional NPPs (Fig. 8b and Figure S6). Comparatively, the lysis of the rapamycin induced group (+ Rap) was significantly lower, with an average lysis value of 0.39% (± 0.26) that was not significantly different to the uninfected control (Fig. 8b and Figure S6). Therefore, induction of *PbclagX* truncation with rapamycin resulted in loss of NPP functionality as parasites were unable to transport the guanidinium solution into RBCs.

## Discussion

According to the current model, the *P. falciparum* NPP is comprised of the RhopH complex, with RhopH1/CLAG3 operating as the surface exposed channel component, structurally supported by RhopH2 and RhopH3 on the cytosolic face. The RhopH complex structure at the membrane has not been solved, but its soluble form shows binding of RhopH2 and RhopH3 directly to *Pf*CLAG3 [71, 73]. It is not known if the remaining *Pf*CLAG proteins also form NPPs that complement *Pf*CLAG3 functionality and hence explains why deletion of *Pf*CLAG3 is not lethal.

Examined here, the lack of protein conservation between *P. berghei* CLAG proteins, including the variance in TMD location and number, the absence of a surface exposed HVR in CLAG9, and their evolutionary divergence, suggests alternative functions for these two CLAG proteins. Indeed, whilst we were successfully able to generate the *Pb*_*clagX/9cntrl*_ line, attempts to generate a *PbclagX* or *Pbclag9* knockout line or a *Pb*_*clagX/9*_ line in which the Ct of *PbclagX* is replaced with *Pbclag9* were unsuccessful*.* These findings support a model where *P. berghei clag* genes have separate, non-complementary functions. The successful generation of both the *Pb*_*clagX/3.1*_ and *Pb*_*clagX/3.1extend*_ lines lend further support to this argument.

Rapid and efficient DNA recombination of the *PbclagX flox* region was induced by rapamycin, resulting in excision of sequences encoding the *Pb*CLAGX Ct functional components. Truncation was detrimental to parasite survival in mice, with parasites dying in the cycle following rapamycin induction. That parasites treated with rapamycin were able to progress to schizonts and invade new RBCs demonstrates that *Pb*CLAGX is not involved in invasion, a role previously ascribed to *Pf*CLAG9 in one study as well as to *Pf*RhopH3 [22, 23, 44]. Rather, parasites were unable to progress past early trophozoite stage and ultimately died. This phenotype could be likened to that seen when *Pf*RhopH2 or *Pb*RhopH2 were conditionally depleted or *Pf*RhopH3 depleted after RBC invasion, resulting in compromised NPPs that led to parasite starvation and death in the trophozoite stage [21–23]. Indeed, NPP functionality following truncation of *Pb*CLAGX was shown to be significantly comprised, tested by the susceptibility to guanidinium lysis, strongly supporting an essential role of *Pb*CLAGX in the NPPs. The death of parasites following the excision of the *PbclagX flox* region also confirms that at least some of the elements within this region, namely the TMD, HVR and RhopH binding regions, do in fact convey functionality. Furthermore, the replacement of said elements with those from *Pfclag3.1* confirms *PbclagX* is indeed the functional ortholog of *Pfclag3*.

It is not currently understood why the elimination of *P. falciparum clag3* does not completely abolish NPP activity and is only critical to growth in PGIM [24–27]. That a complete *Pfclag3* knockout was only able to exhibit a decreased growth phenotype when cultured in a PGIM modified medium [25, 26] could indicate that this medium does not fully mimic the nutrients found in human serum. Alternatively, the non-lethal phenotype could also indicate that there is some functional, albeit incomplete, complementation with the other CLAG paralogs. This could be because there is either a lower abundance of *Pf*CLAG2 and/or *Pf*CLAG8 in comparison to *Pf*CLAG3 to form NPPs, or that the RhopH complexes containing other *Pf*CLAG paralogs have an inferior transport capacity in PGIM compared to *Pf*CLAG3.

## Conclusion

Shown here, *P. berghei* CLAGX essentiality could be definitively established and linked to the NPPs functionality utilising the *Pb*_*clagX/flox*_ line, providing the first functional evidence for CLAG essentiality in an in vivo model. Our findings highlight the critical role that a *Pfclag3* orthologue plays to in vivo growth and survival of parasites and supports the continued investigation of the RhopH complex as a potential therapeutic target, given it is parasite specific, and an essential protein complex. Although the surface exposed HVR region of CLAG proteins are variable, and this may be a reasonable concern for targeted drug design, there are highly conserved regions within the CLAG proteins, such as the RhopH complex binding regions, that could be targeted. Moreover, targeting these regions may impact the function of all the CLAG proteins, including CLAG9, which has a non-overlapping function.

## Supplementary Information

Additional file 1.

## Data Availability

The datasets used and/or analysed during the current study are available from the corresponding author on reasonable request.

## Abbreviations

aa: Amino acids
BSA: Bovine serum albumin
*clag*: Cytoadherence linked asexual protein
cntrl: Control
Ct: C-terminal
DAPI: 6-diamidino-2-phenylindole
DMSO: Dimethyl sulfoxide
*flox*: Flanked by *loxP*
gDNA: Genomic DNA
gRNA: Single-guide RNA
HA: Haemagglutinin
HB: Homology box
Hct: Haematocrit
hDHFR: Human dihydrofolate reductase
hpr: Hours post rapamycin-induction
HVR: Hypervariable region
Int: Integration
IP: Intraperitoneal
iRBCs: Infected red blood cells
IV: Intravenous
MFS: Malaria freezing solution
MT: Mouse tonicity
Nt: N-terminal
NPPs: New permeability pathways
OD: optical density
*Pb*ANKA*/PbA*: *Plasmodium berghei* ANKA
*P*. *berghei*/*Pb*: *Plasmodium* *berghei*
*P*. *falciparum*/*Pf*: *Plasmodium* *falciparum*
PGIM: *Plasmodial* surface anion channel growth inhibition media
PI: Protease inhibitors
PYR: pyrimethamine
Rap: Rapamycin
RBCs: Red blood cells
RPMI: Roswell Park Memorial Institute media
RT: Room temperature
spp.: Species
TMD: Transmembrane domain
UTR: Untranslated region
3′: Three prime
5′: Five prime
