# Supplementary material for: Utilisation of an in vivo malaria model to provide functional proof for RhopH1/CLAG essentiality and conserved orthology with P. falciparum
Source: J Biomed Sci. 2025 Feb 3;32:13. doi: 10.1186/s12929-024-01105-7 (PMC11789411; doi:10.1186/s12929-024-01105-7)
Supplement: Supplementary file 1 — Additional file 1. [file 12929_2024_1105_MOESM1_ESM.docx]

**
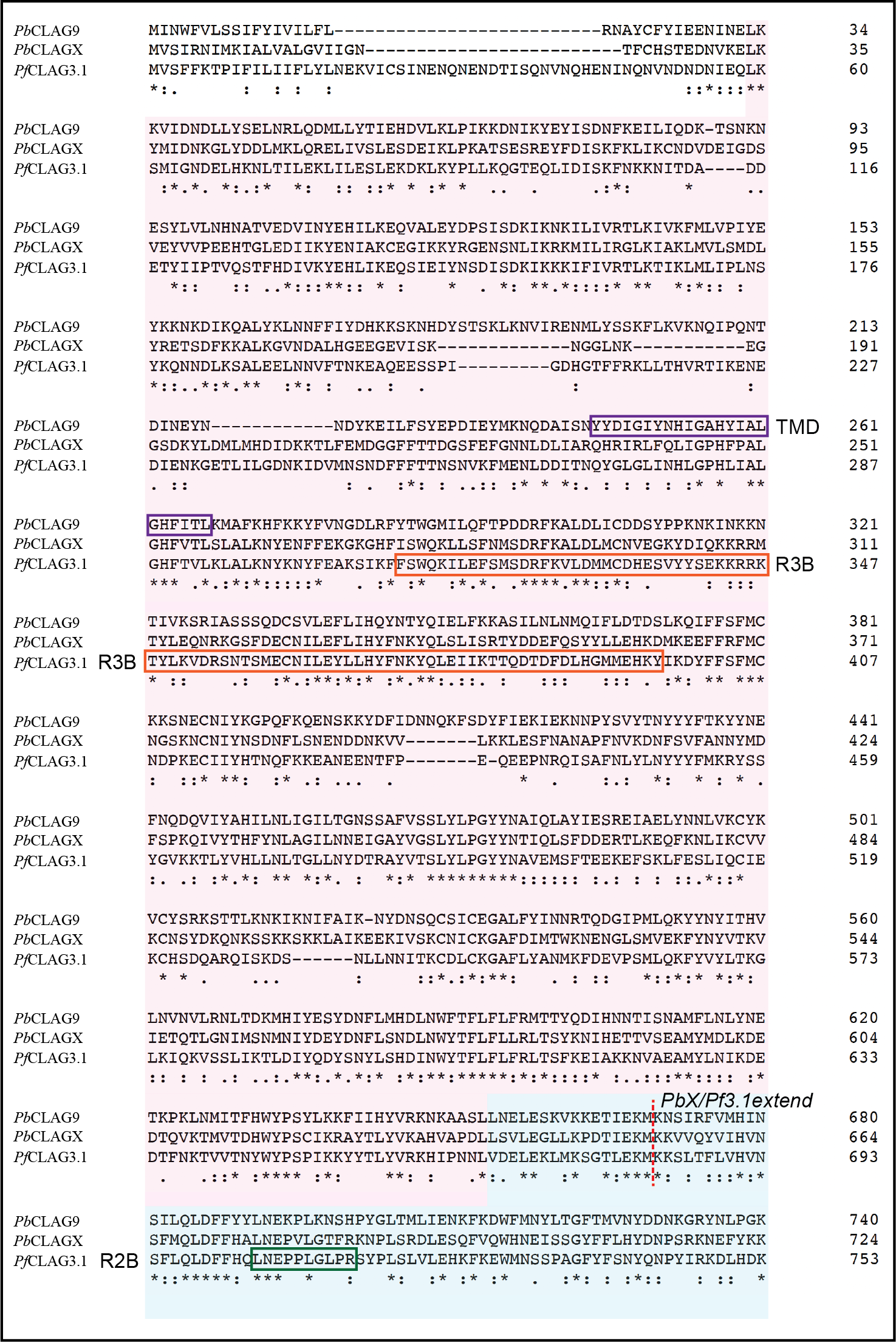
**

**
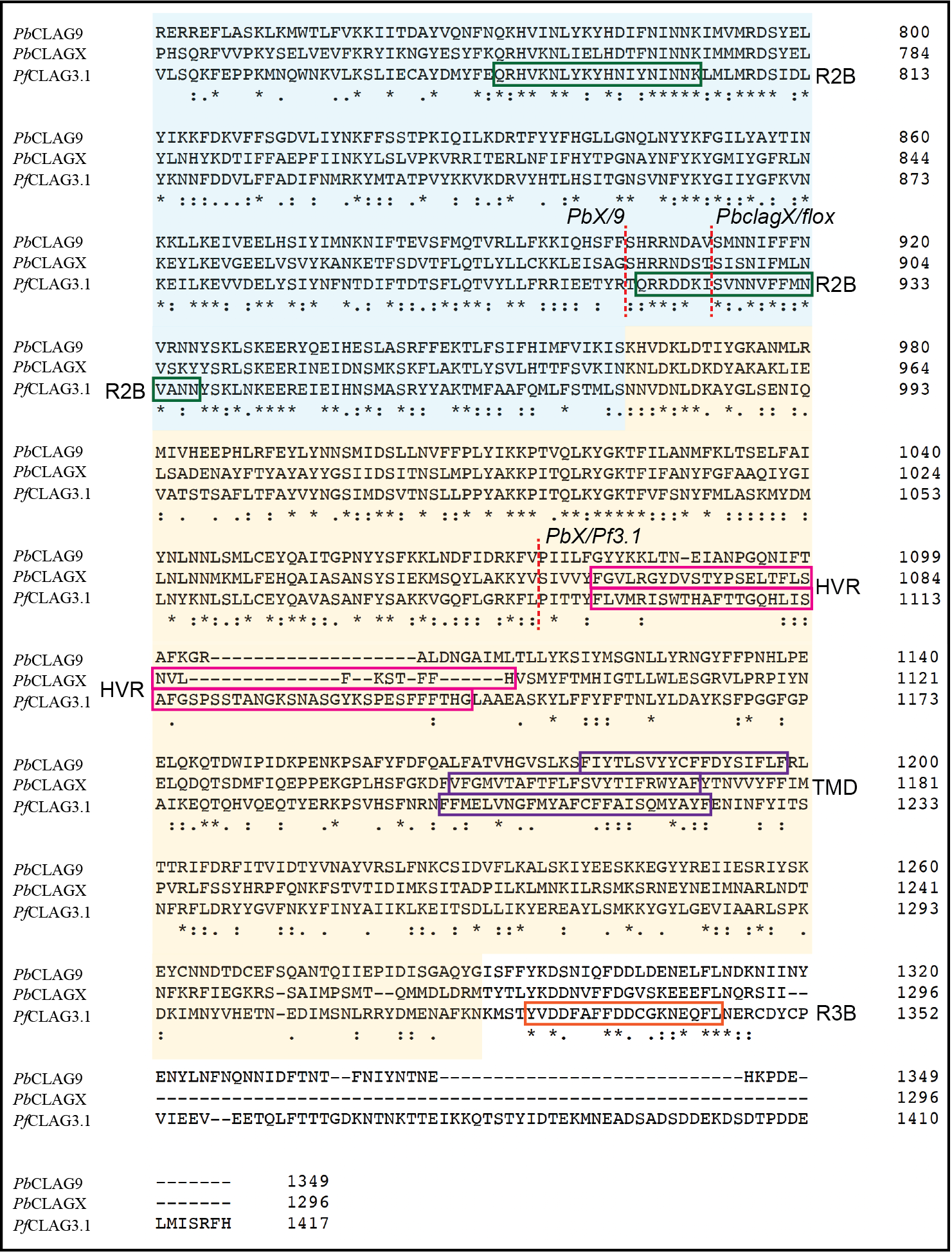
Supp Figure 1. Alignment of *P. berghei* CLAGX and CLAG9 and *P. falciparum* CLAG3.1.** Multiple sequence alignment (Clustal-Omega) of the CLAG proteins focused on in this study. *Pf*RhopH2 (R2) and *Pf*RhopH3 (R3) binding regions (B) boxed in green and orange respectively, as well as structural regions of *Pf*CLAG3 revealed from the cryo-EM structure of the soluble RhopH complex [[71](#_ENREF_71), [73](#_ENREF_73)] are indicated, the latter highlighted as follows; pink: Nt sphere, blue: RhopH2 bridge, yellow: Ct bundle/Helical bundle. The predicted HVR (boxed in pink) and TMD (boxed in purple) are also displayed (Ho et al., 2021). Red segmented lines indicate starting regions used in the allelic replacement and knockout constructs. (*) Conserved; (:) conservative; (·) semi-conserved; (gap) no conservation.


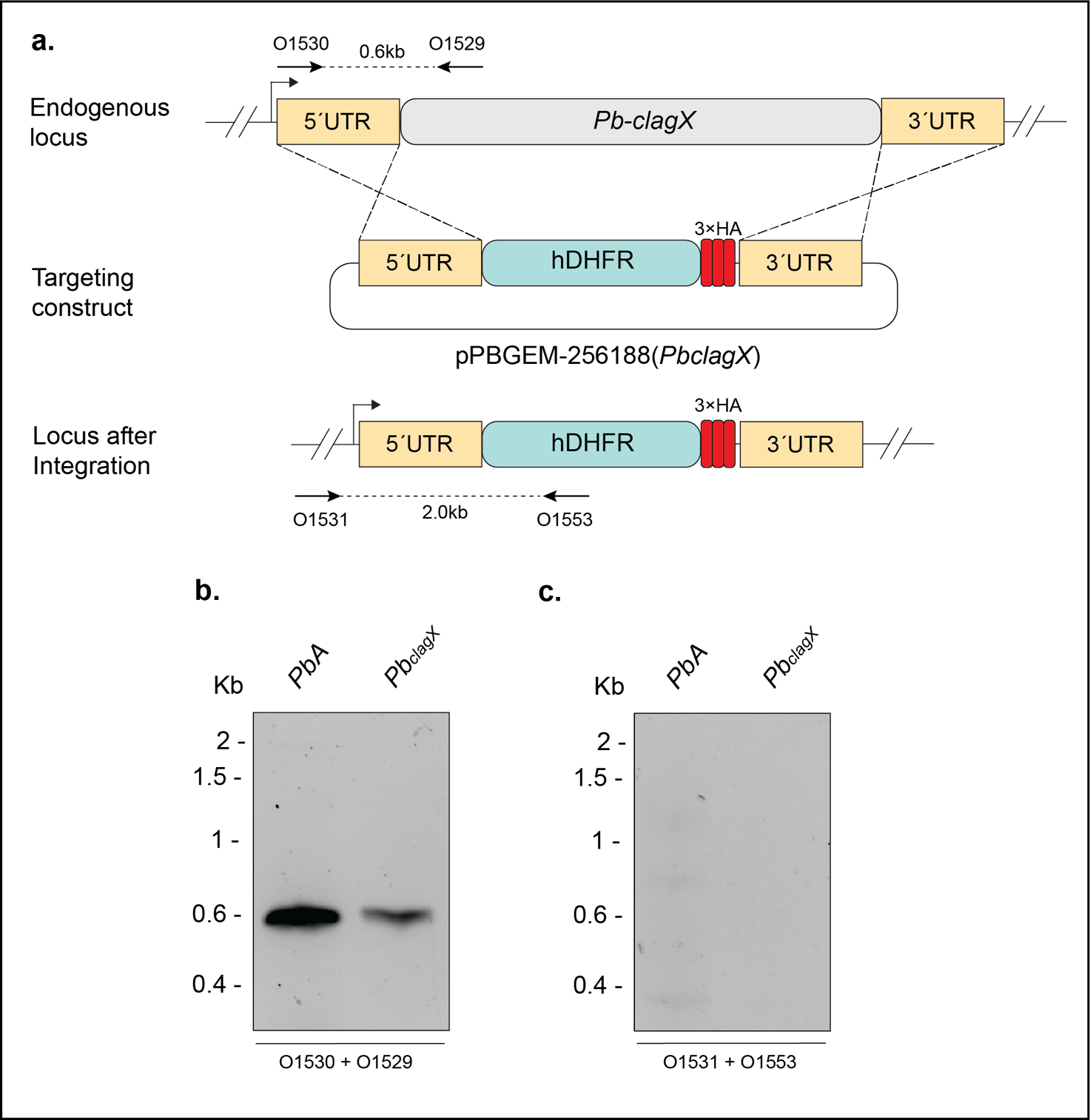
**Supplementary Figure 2. *PbclagX*** **knockout schematic and integration PCRs. (a)** Construct design for knockout of the *clagX* gene in *P. berghei* utilizing the Sanger construct PBGEM_256188 [[75](#_ENREF_75)]. Double cross-over homologous recombination in the 5´ and 3´UTR facilitate the replacement of the *clagX* gene with the hDHFR drug selectable marker C-terminally tagged with 3$\times$HA epitopes. **(b)** PCR of gDNA from parasites retrieved following transfection (*PbclagX*) or from WT parasites (*PbA*) using primers to detect a wildtype locus, or **(c)** an integrated locus.

**
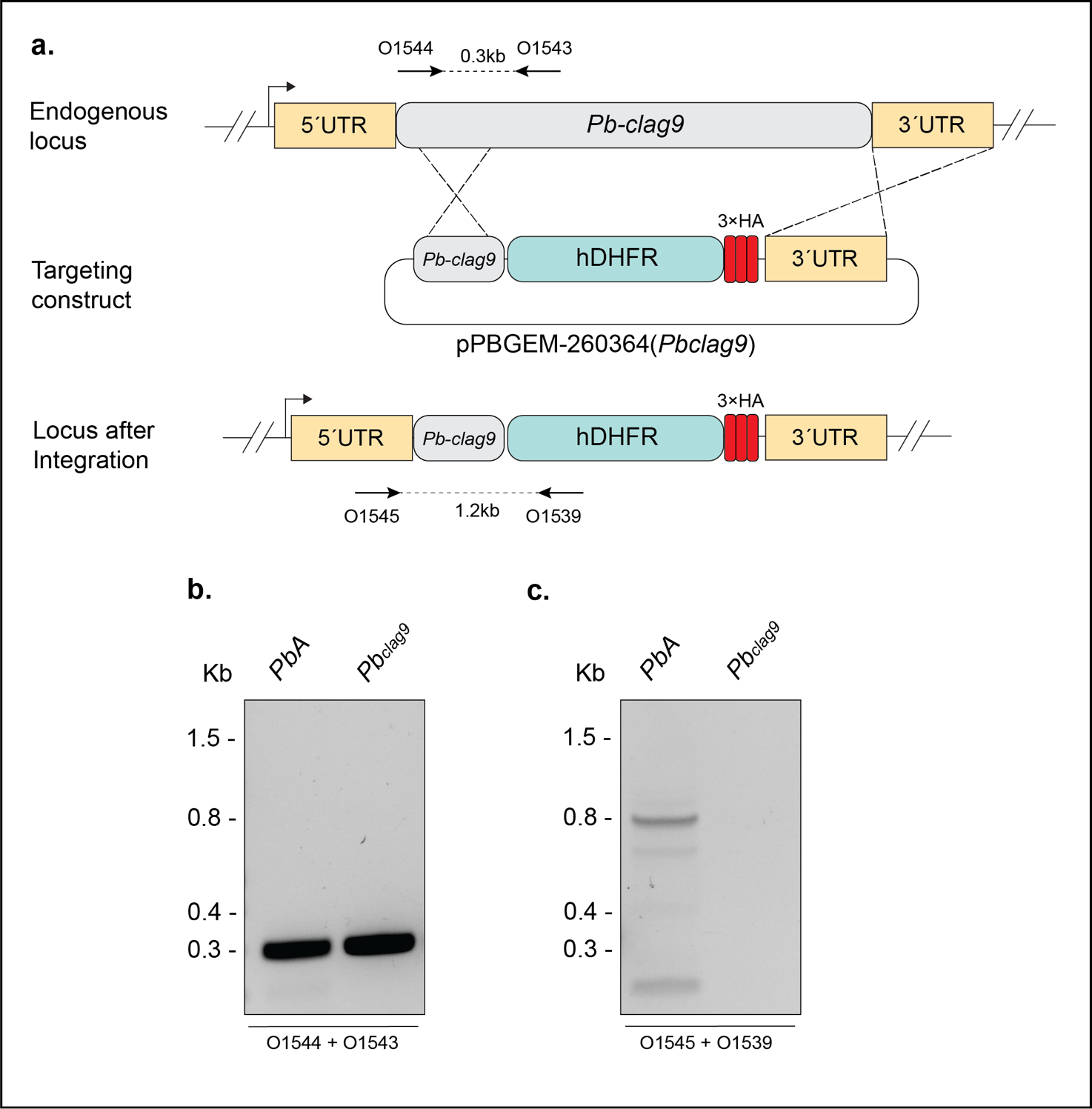
**

**Supplementary Figure 3. *Pbclag9*** **knockout schematic and integration PCRs. (a)** Construct design for knockout of the *clag9* gene in *P. berghei* utilizing the Sanger construct PBGEM_260364 [[75](#_ENREF_75)]. Double cross-over homologous recombination in the 5´-coding region and 3´UTR facilitate the replacement of the *clag9* gene with the hDHFR drug selectable marker C-terminally tagged with 3$\times$HA epitopes. **(b)** PCR of gDNA from parasites retrieved following transfection (*Pbclag9*) or from WT parasites (*PbA*) using primers to detect a wildtype locus, or **(c)** an integrated locus.

**
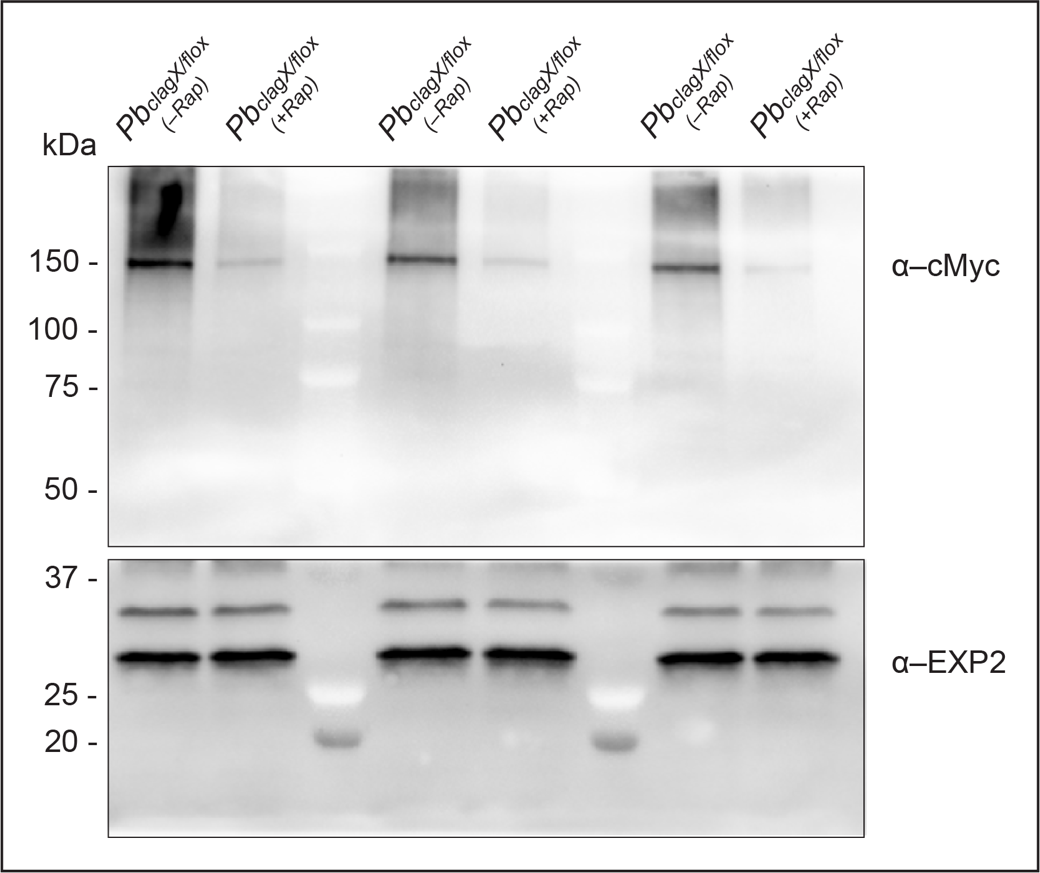
**

**Supplementary Figure 4. Western blot to quantitate *PbclagX* protein knockdown.** Biological replicates of *PbclagX/flox* parasites harvested from mice (n=3) and cultured *in vitro* in the presence of DMSO vehicle control (–Rap) or rapamycin (+Rap) to schizont stage of development. Parasite lysate was analysed by western blotting. The expected size of cMyc tagged CLAGX is 152 kDa, whereas EXP2 (32 kDa) served as the loading control. The amount of CLAGX remaining after rapamycin induction was calculated relative to the ­­­loading control.

**
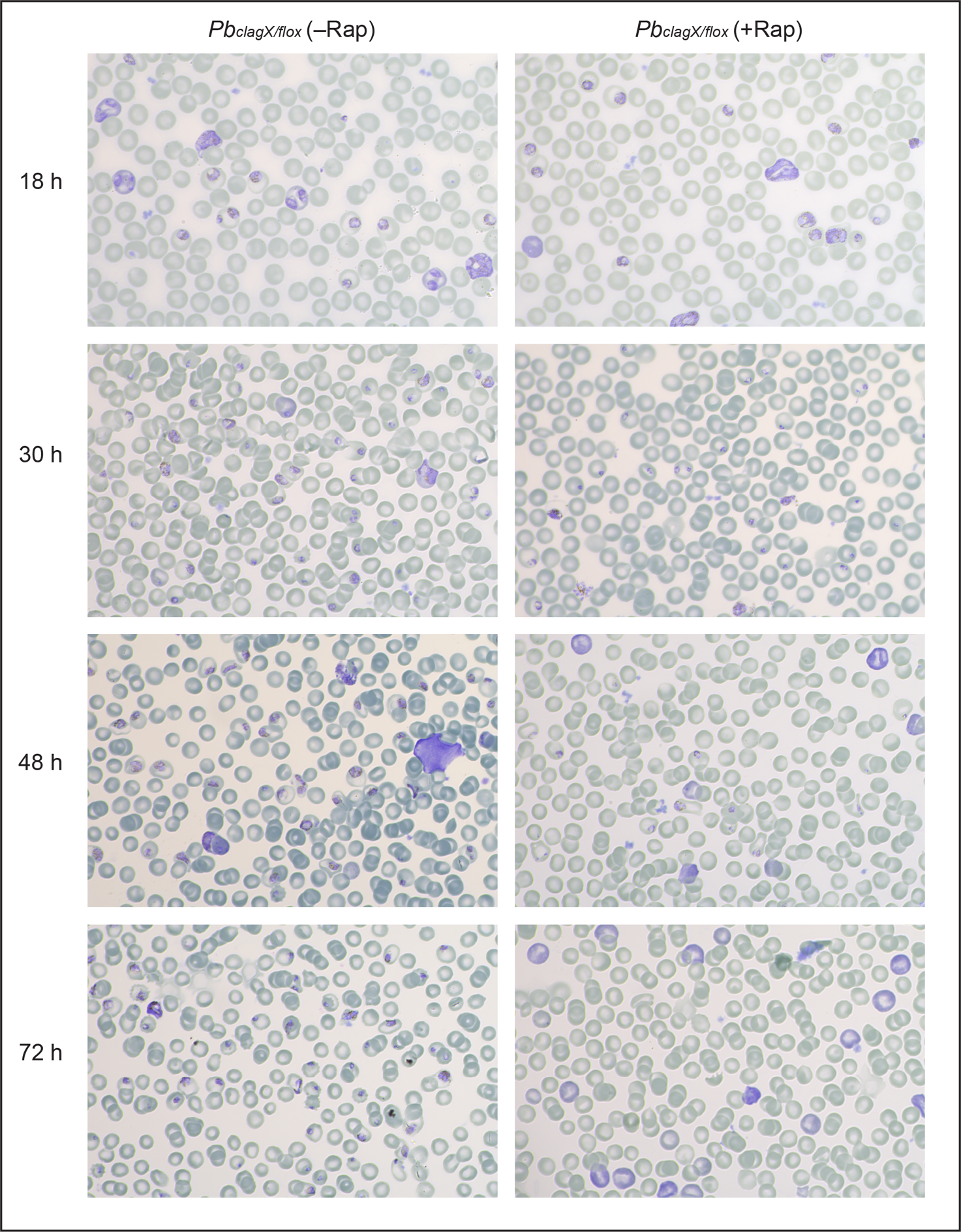
Supplementary Figure 5. *In vivo* knockout of *PbclagX* affects parasite development and survival.** Mice infected with the *PbclagX/flox* parasite line were either administered DMSO vehicle control (–Rap) or 4 mg/kg rapamycin (+Rap) (n=5). Giemsa stains used to monitor parasite development over 72 h following rapamycin induction. Panels shown are representative of those shown in Figure 7.

**
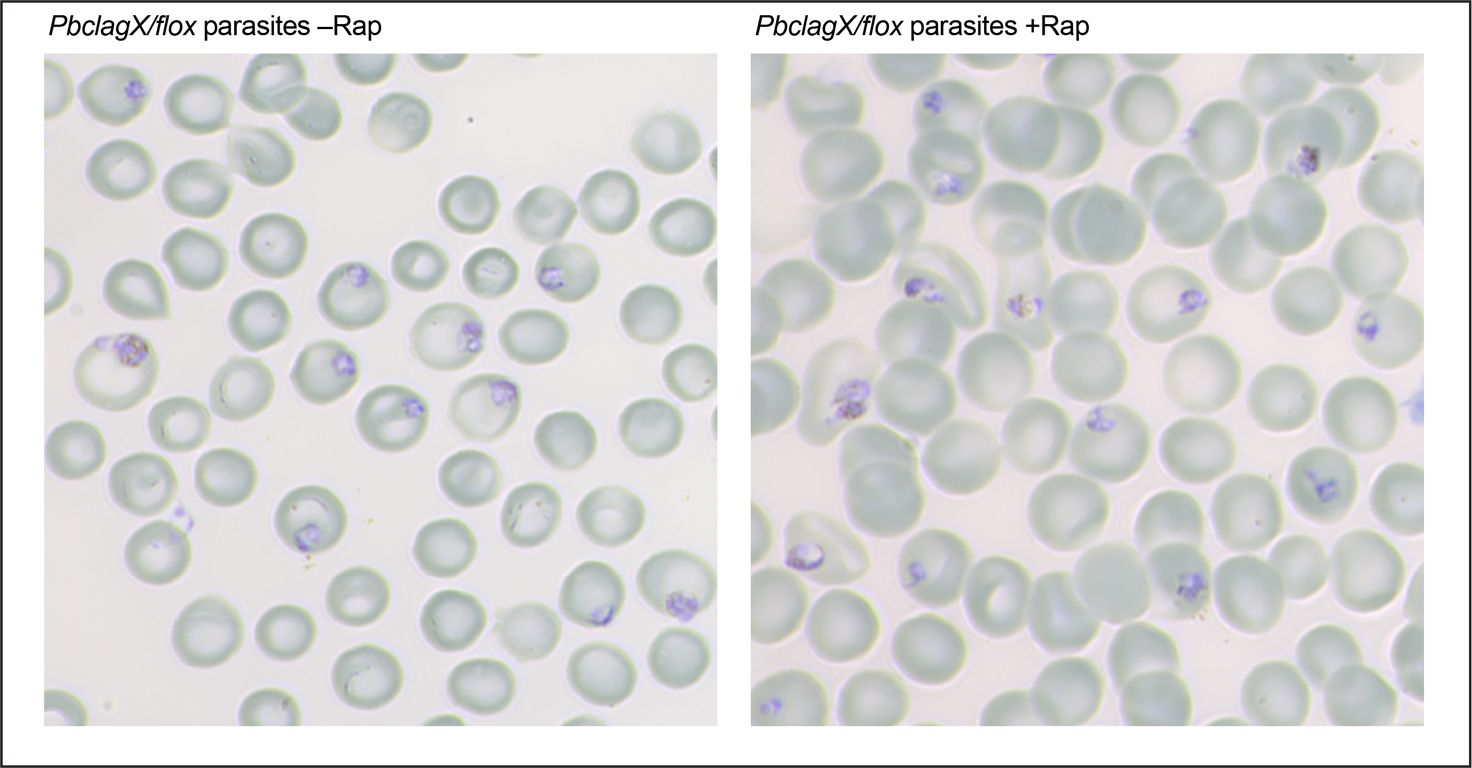
**

**Supplementary Figure 6. Representative Giemsa image of *PbclagX/flox* parasites harvested for NPP assessment.** Mice infected with *PbclagX/flox* parasites were either injected with vehicle control (–Rap) or 4 mg/kg rapamycin (+Rap) to induce *clagX* excision. Approx. 30 hpr parasites were harvested from mice and susceptibility to guanidinium chloride was examined (n=3). Panels shown are representative of those shown in Figure 8.

**
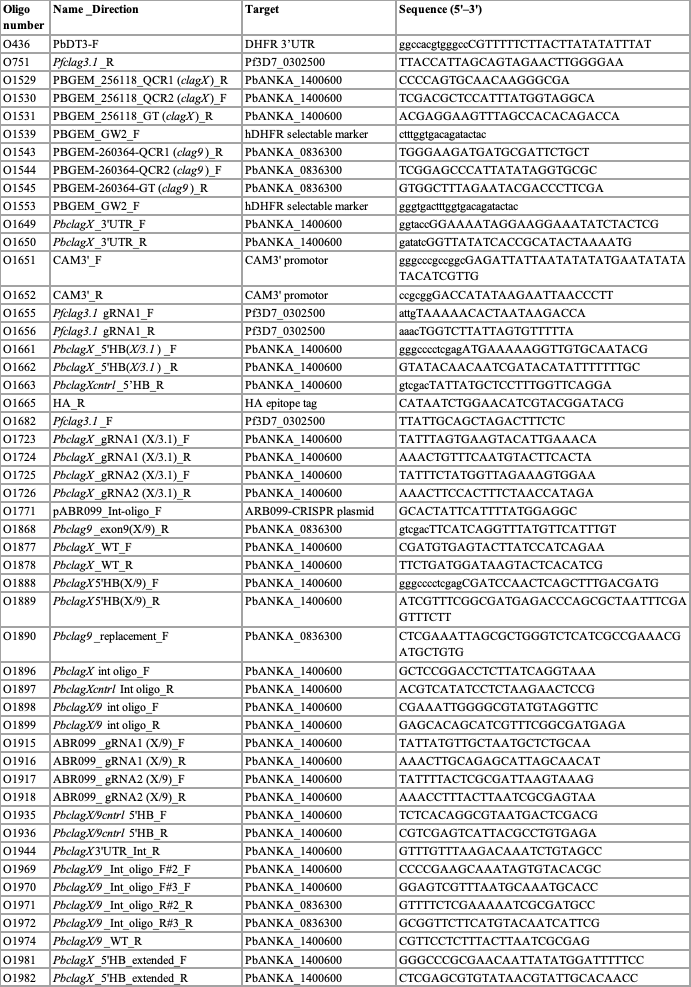
Supplementary Table 1. Oligonucleotides used in this study**

**
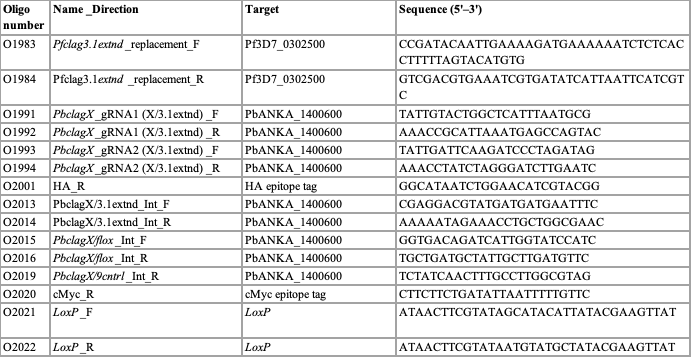
**
